# Transcriptomic investigation of agonistic behaviors of boxer shrimps (*Stenopus* species): insights into the potential neural signaling roles of dopamine and acetylcholine

**DOI:** 10.1186/s12864-026-12899-1

**Published:** 2026-05-08

**Authors:** Terance Ho Him Wong, Lai Him Chow, Ziwei Wu, Tom Kwok Lun Hui, Ling Ming Tsang

**Affiliations:** 1 Simon F.S. Li Marine Science Laboratory, School of Life Sciences, The Chinese University of Hong Kong, Shatin, Hong Kong SAR China; 2 Department of Microbiology and Immunology, The Peter Doherty Institute for Infection and Immunity, University of Melbourne, Victoria, Australia

**Keywords:** Agonistic behaviors, Crustaceans, Genetics, Hormones, Neuroendocrinology, Transcriptome

## Abstract

**Background:**

Agonistic behaviors are crucial and common among animals due to their importance in securing an individual’s fitness, and neural signaling molecules are known to mediate these behaviors. *Stenopus*, a genus of shrimp-like decapod crustaceans characterized by a pair of enlarged pereiopods, exhibits prominent agonistic behaviors when encountering conspecifics of the same sex owing to its monogamous social structure. These shrimps represent another potentially excellent model organism for investigating the neural signaling basis of agonistic behaviors in crustaceans aside from traditional models. Yet, their underpinning molecular aspects have never been studied. Using *S. hispidus* and *S. cyanoscelis* as representatives, the present study is the first that systematically examines the genetics of agonistic behaviors in *Stenopus*. Three organs, including (1) antennae + antennules, (2) central nervous system, and (3) eyestalk ganglia, were RNA-sequenced to identify the differentially expressed genes (DEGs) and pathways potentially conserved in winners and losers of *Stenopus* after fighting interactions.

**Results:**

Our results suggested that *Stenopus* agonistic interactions might be systemic activities involving the simultaneous modulation and interplay of multiple signaling cascades, organismal systems, and metabolic pathways. In particular, winners and losers typically exhibited enriched gene ontologies involved in neural signaling, and sensory and behavioral processes. Regarding enriched pathways, while those related to glycan biosynthesis and metabolism were enriched in winners, cholesterol metabolism and one-carbon pool by folate were enriched in losers. These different sets of pathways suggested that while fighting interactions in *Stenopus* were injurious to both combatants, the damage in losers appeared to be more traumatic. Furthermore, four neural signaling systems, including dopamine, acetylcholine, octopamine, and glutamate, were identified as potentially major mediators of agonistic behaviors and fighting interactions in both *Stenopus* species, with the first two appearing to be relatively more important. A comparison of the neural signaling systems involved in mediating aggression among pan-crustaceans suggested that *Stenopus* appeared to stand out by its seemingly major reliance on dopamine and acetylcholine, as opposed to the primarily serotonin-based regulation of aggression observed in most examined pan-crustaceans.

**Conclusions:**

The different metabolic responses between winners and losers in *Stenopus* highlight the profound, asymmetric physiological costs of social conflict at the molecular level. Furthermore, their unique reliance on dopamine and acetylcholine reveals diverse evolutionary trajectories in the neuroendocrine regulation of aggression, providing new insights into the current paradigms of invertebrate social behavior.

**Supplementary Information:**

The online version contains supplementary material available at 10.1186/s12864-026-12899-1.

## Background

Agonistic behaviors are ubiquitous and crucial among conspecifics for the competition of limited resources, such as shelters and mates, to gain better opportunities for survival, growth, and reproduction [1]. As agonistic interactions are typically energetically costly and potentially injurious to both contestants, contestants often signal aggression via different sensory cues in advance, such as visual, olfactory, and tactile cues. Upon assessing the opponent’s fighting abilities (e.g., body mass and aggressive motivation) relative to its own, one contestant may choose to retreat, or both may decide to engage in fighting [2, 3]. In the latter situation, upon the establishment of a winner-loser relationship, winners typically continue to exhibit aggressive behaviors and will gain greater access to the resources, whereas losers will display appeasement behaviors and have to retreat to avoid further injuries [4].

Neural signaling molecules, such as neurohormones, neurotransmitters, and neuromodulators [5], are all known to mediate animal physiologies and behaviors, including agonistic behaviors [6–8]. Among invertebrates, incredible progress has been made in understanding the genetic and neural signaling basis of agonistic behaviors in insects, especially fruit flies of the genus *Drosophila*, the model organisms for arthropods and invertebrates in general [9]. A number of these neural signaling molecules, such as serotonin, dopamine, octopamine, acetylcholine, glutamate, gamma-aminobutyric acid (GABA), tachykinin, and neuropeptide F, have been found to be crucial in mediating their agonistic behaviors [9–12]. Co-regulation and signaling of aggression by these molecules is also not unusual [13–15]. The mediation of agonistic behaviors by these molecules has also been similarly reported in other insect taxa, such as crickets [16, 17], wasps [18, 19], beetles [20], and ants [21], suggesting their importance and function among insects in general.

Also being pan-crustaceans (i.e., a subgroup of arthropods), crustaceans are underrepresented in the study of aggression regulation compared to insects. While some neural signaling molecules, such as neurohormone SIFamide and crustacean hyperglycemic hormone, appear to specifically mediate agonistic behaviors in crustaceans [22, 23], others are generally similar to those documented in insects (e.g., octopamine, serotonin, and glutamate), identified mainly based on common crustacean models, such as crayfish, lobsters, and some species of shrimps (e.g., giant freshwater prawn) and crabs (e.g., Chinese mitten crab) [24–27]. While some neural signaling molecules are conserved between crustaceans and insects, their functional roles could vary significantly across taxa, especially when they are more distantly related. An example of that is the neuromodulator, serotonin. While Dierick and Greenspan [13] and Alekseyenko et al. [28] independently demonstrated that increased serotonin level resulted in an escalation of aggression in fruit flies, Doernberg et al. [25] surprisingly discovered that both elevating and depleting serotonin increased fighting tendencies in American lobsters. Yet, Yang et al. [26] reported that serotonin level in Chinese mitten crabs increased after a fight. These studies demonstrate that more species need to be investigated to understand the variability of the genetic and neural signaling basis of agonistic behaviors in crustaceans. This will improve our understanding of the variability and conservation of biological and physiological processes among pan-crustaceans as well as within crustaceans, shedding light on the evolution of neural signaling.

In accordance with recent advocates of expanding the use of invertebrate animal models [29, 30], we hereby aim to establish *Stenopus* shrimps (order Decapoda, infraorder Stenopodidea) as another model organism for studying the genetic and neural signaling basis of agonistic behaviors in crustaceans to complement the findings from traditional crustacean models. *Stenopus*, characterized by an enlarged third pair of pereiopods modified into chelipeds, exhibits prominent agonistic behaviors [31], probably attributed to its monogamous social structure and territoriality [32, 33]. Despite its well-known aggressiveness, transcriptomic and nuclear genomic information have remained scarce for this genus [34], and the neurochemical basis of its agonistic behaviors has never been examined. Therefore, using *S. hispidus* (Olivier, 1811) and *S. cyanoscelis* (Goy, 1984) as representatives, the present study aims to find out the genetic and neural signaling basis, as well as the underpinning physiological mechanisms potentially involved in mediating the agonistic behaviors of *Stenopus* via RNA-sequencing and subsequent differential gene expression and enrichment analyses. Previous animal aggression regulation studies focused considerably on winners and their aggressive behaviors; however, losers and their appeasement behaviors were largely neglected [35–37]. Therefore, the present study also attempts to investigate the behavioral genetics of the entire dominant-subordinate system to better understand the full picture. The transcriptomic profiles and physiological mechanisms of the shrimps’ peripheral sensory organs, which are also the drivers of *Stenopus* agonistic behaviors and fighting interactions (i.e., antennae and antennules combined) [31], behavior-controlling organ (i.e., central nervous system (CNS)) [38], and neurohemal centers (i.e., eyestalk ganglia) [39] are compared between (1) winners and controls, (2) losers and controls, and (3) winners and losers, to identify potentially major neural signaling systems and physiological pathways involved in fighting in winners and losers of each of the two species, and *Stenopus* overall, providing valuable insights into conflict resolution in this genus. By establishing and subsequently comparing the neural signaling profile of *Stenopus* with that of other crustaceans and insects previously examined for the regulation of aggression, the present study will shed light on the variability in and/or conservation of neural signaling systems mediating agonistic behaviors among crustaceans and pan-crustaceans.

## Methods

### Collection and maintenance of shrimps

Non-ovigerous, sexually mature females with advanced ovaries (i.e., an ovarian maturation stage in *Stenopus* in which the ovaries are bluish green in color [40]) of *Stenopus hispidus* and *S. cyanoscelis* were purchased from aquaria in Hong Kong, China, and the shrimps were maintained individually in an isolated, 10 cm × 10 cm × 10 cm cubic tank in a flow-through seawater system in the laboratory for 10-day acclimatization before being used in experiments. The salinity and temperature of the system was kept in a range between 33$\:-$35 ppt and 20$\:-$22$\:℃$, respectively, and the shrimps were fed fresh pieces of clam tissue twice a week during the acclimatization period. Only females of both species were utilized in this study, because sexing of females could easily be performed by observing the presence of bluish green ovaries, as opposed to males that require microscopic examination, which would easily stress the shrimps out before experiments and might cause a loss of appendages. Nevertheless, using females alone in the present study should remain generally representative of this genus, because both sexes are typically equally aggressive towards conspecifics of the same sex [32, 33], and females are typically as aggressive as males due to the monogamous social structure in *Stenopus*, which has no apparent sexual dimorphism of cheliped weaponry [31].

### Pairing of shrimps and treatment tanks preparation

**Fig. 1:**
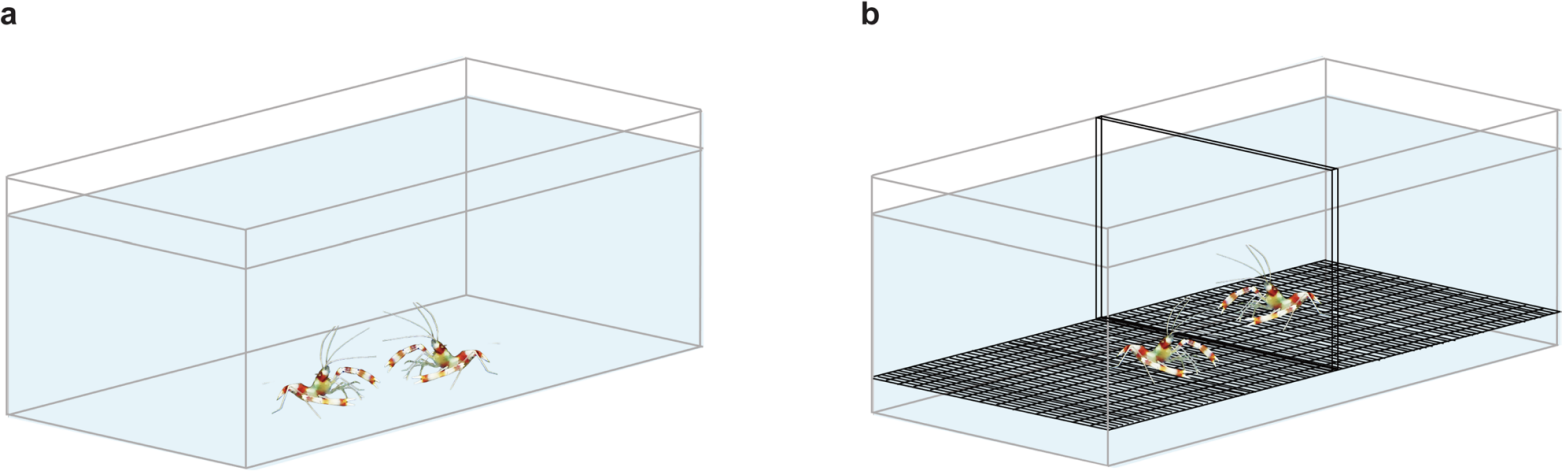
**a** treatment tank and (**b**) control tank employed in the present study for finding out the differentially expressed genes and neural signaling systems potentially mediating Stenopus agonistic behaviors and fighting interactions

A total of ten *S. hispidus* and ten *S. cyanoscelis* individuals were utilized in the present study. Two similar-sized conspecifics (i.e., within 8% size differences) of the same species were paired based on their body length, measured from the tip of rostrum to the end of telson using an electronic, water-proof caliper. Subsequently, two of the five shrimp pairs were stochastically allocated to the control group, and the remaining three pairs to the treatment group, for both species. As we demonstrated that tactile cues, rather than visual and olfactory cues, played a prerequisite role in inducing the agonistic behaviors of *Stenopus* shrimps in our previous study [31], the treatment tank for the present study was designed to allow physical contact among shrimps. The tank was 31 cm × 17 cm × 20 cm (length × width × height) large and had no physical barriers, such that tactile contact, as well as visual and olfactory detection between the two contestants were permitted (Fig. 1a). The control tank, on the other hand, was structurally modified from the treatment tank. A nylon net was glued horizontally, several centimeters above the bottom of the tank to produce a lower and an upper compartment, and the upper compartment was further divided into two equally-sized partitions by a transparent acrylic plate (Fig. 1b). Consequently, the two individuals on the upper compartment could “see” each other via the acrylic plate, and could exchange chemical signals via the lower compartment (as confirmed by the diffusion of dyes across the lower compartment in a preliminary test), but could not touch each other. In line with our previous study [31], we observed that agonistic behaviors were largely absent from the two individuals inside the control tank even when both visual and olfactory detection were allowed, showing that this control tank design was appropriate for the present study.

Before the commencement of the experiment, the two individuals were separated in the tank for 15 min of acclimatization by inserting a black, opaque acrylic plate in the middle of the tank to impede any prior contact between the two individuals. However, olfactory detection in the control tank could not be impeded during the acclimatization period, as prevention of such would require placing a barrier between the shrimps and the nylon net, and subsequent removal after acclimatization would inevitably cause significant human disturbances. According to our previous study [31], the dominant-subordinate relationship between the two individuals of *Stenopus* might switch (though rarely) during fighting and became fixed only when the submissive individual lost a cheliped. Therefore, in this study, the individual that ultimately lost its cheliped after fighting was defined as the loser, whereas its opponent was thereby the winner. This situation was generally observed to occur around (mean $\:\pm\:\:$SD) 24 $\:\pm\:$ 6 min in both *Stenopus* species in this study (additional information file Table S1). To ensure sufficient time for genes to be transcribed, which typically takes approximately 6 to 10 min according to *Drosophila* and mammalian cell line studies [41, 42], an additional 10 min of interaction between the winner and loser individual was permitted after the loser had lost its cheliped, leading to a final experimental duration of approximately (mean $\:\pm\:\:$SD) 34 $\:\pm\:$ 6 min (additional information file Table S1).

### Total RNA extraction and transcriptome sequencing

Four organs, including antennae, antennules, CNS, and eyestalk ganglia were harvested from experimental individuals for RNA extraction. Due to the potential functional overlap between antennae and antennules in triggering *Stenopus* agonism [31], the RNA solutions from these two organs were pooled and treated as one organ in this study, resulting in a final of three RNA samples (i.e., antennae + antennules, CNS, and eyestalk ganglia) analyzed per individual. To maintain consistency in sample size with the treatment group, only three of the four individuals from the control group, along with three winners and three losers from three fighting pairs in the treatment group, were dissected. Total RNA was extracted from the harvested organs using TRIzol reagent Invitrogen following the standard protocol. The quality and quantity of a total of 54 RNA samples (i.e., 9 individuals × 3 organs × 2 species) were confirmed by 1.2% agarose electrophoretic gel in conjunction with Agilent 2100 Bioanalyzer system and Nanodrop assay, and were subsequently sent to Beijing Genomics Institute (BGI), Hong Kong, China, for RNA-sequencing. Briefly, mRNA molecules with poly(A) tail were enriched from total RNA using oligo d(T) beads, and were then fragmented to synthesize first strand cDNA, followed by second-strand cDNA synthesis using dUTP nucleotides for strand-specific library construction. Adapter sequences were then ligated to both sides of the cDNA of both strands for PCR amplification and subsequent paired-end sequencing on DNA nanoball sequencing (DNBSEQ) platform to produce ~ 10 Gigabyte (Gb) 150-base pair (bp) raw reads per sample. The sequencing quality of each sample was evaluated by Phred quality score for filtering out low-quality bases.

### *De novo* transcriptome assembly and functional annotation

Quality of the raw reads was examined by FastQC v0.11.9 [43], and then the adapter and contaminant sequences were removed by Cutadapt v4.2 [44] and KrakenUniq v1.0.3 [45], respectively, followed by re-pairing of forward and reverse reads using Pairfq v0.17.0 [46]. As no reference genome is available for *Stenopus* shrimps, upon the quality confirmation of the resulting clean reads by FastQC again [43], *de novo* transcriptome assembly was performed to recover full-length transcripts while low-quality bases were simultaneously removed, using Trinity v2.15.1 with the Trimmomatic option [47]. Qualities of the transcriptomes of both *S. hispidus* and *S. cyanoscelis* were examined based on transcript N50 contig length [48] and scores from Benchmarking Universal Single-Copy Orthologs (BUSCO) v5.4.7 [49]. Open reading frames (ORFs) within the transcripts were then extracted by TransDecoder v5.5.0 [50], and the resulting predicted protein-coding sequences were annotated using DIAMOND BLAST v2.1.8 [51], searching against six databases, including National Center for Biotechnology Information (NCBI) non-redundant protein database [52], SWISS-PROT [53, 54], Pfam [55], Evolutionary genealogy of genes: Non-supervised Orthologous Groups (eggNOG) v5.0 [56], Gene Ontology (GO) [57, 58], and Kyoto Encyclopedia of Genes and Genomes (KEGG) v108.1 [59], with a filtering threshold of E-value < 10^− 4^. All annotations of the unigenes were systematically integrated using Trinotate v3.1.1 [60].

### Transcript quantification, differential gene expression, and enrichment analyses

Transcript abundance of the three organs for the three control individuals, three winners, and three losers of both species was calculated using RSEM [61], and principal component analysis (PCA) was conducted to visualize their expression patterns (additional information file Fig. S1). Differential gene expression analysis was then performed for winner vs. control, loser vs. control, and winner vs. loser for all three organs, using DESeq2 [62], with filtering criteria of |log_2_FC| > 1.5 and p-value < 0.05. The comparison between winners and losers is important for examining whether there were significant expression level differences for the same differentially expressed genes (DEGs) that were mutually upregulated or downregulated in both groups when compared to control individuals. The resulting DEGs from all three organs for all three pairwise comparisons of both species were then subjected to GO and KEGG enrichment analyses to find out the underpinning physiological pathways, using clusterProfiler v4.12.5 [63] (additional information file Tables S2-S4). Mutual DEGs, GOs, and KEGG pathways between the two *Stenopus* species were then identified and were considered potentially major and representative in mediating the agonistic behaviors of *Stenopus*.

## Results

### Transcriptome assembly results

Despite the lack of a reference genome for *Stenopus*, the transcriptomes of both studied species had high BUSCO completeness scores of at least 99.0%, indicating that they were relatively complete. The average transcript contig N50 value was 678 bp for *S. hispidus* and 611 bp for *S. cyanoscelis* (Table 1).

**Table 1:** Transcriptome assembly results of *S. hispidus* and *S. cyanoscelis*

	*S. hispidus* (*N* = 9 shrimps)	*S. cyanoscelis* (*N* = 9 shrimps)
Total trinity transcripts	1,519,695	2,799,553
Total trinity genes	1,169,992	2,275,179
Average transcript contig length (bp)	539	529
Average transcript contig N50 (bp)	678	611
BUSCO assessment results	C:99.2% [S:64.1%, D:35.1%], F:0.7%, M:0.1%, n:1013	C:99.1% [S:55.7%, D:43.4%], F:0.7%, M:0.2%, n:1013

*bp* base pair

### Overview of the DEGs in *S. hispidus* and *S. cyanoscelis*

The total number of DEGs in *S. hispidus* and *S. cyanoscelis* was 28,145 and 37,454, respectively (additional information file Figs. S2, S3). Organ-wise, the proportion of upregulated and downregulated DEGs in CNS and eyestalk ganglia for all three pairwise comparisons (i.e., winner vs. control, loser vs. control, and winner vs. loser) of both species was generally similar, exhibiting a ~ 1:1 ratio (additional information file Fig. S4). However, in antennae + antennules, the proportion of DEGs was largest in the winner vs. control comparison for *S. hispidus*, with more upregulated than downregulated ones. In contrast, for *S. cyanoscelis*, the largest proportion of DEGs in antennae + antennules was observed in the loser vs. control comparison, with both up- and downregulated ones being proportionally similar. Additionally, PCA revealed similarities in expression patterns of the DEGs among the three winners, among the three losers, and among the three control individuals for all three organs in both species (additional information file Fig. S1).

### GO and KEGG enrichment analysis results of *S. hispidus*

**Fig. 2:**
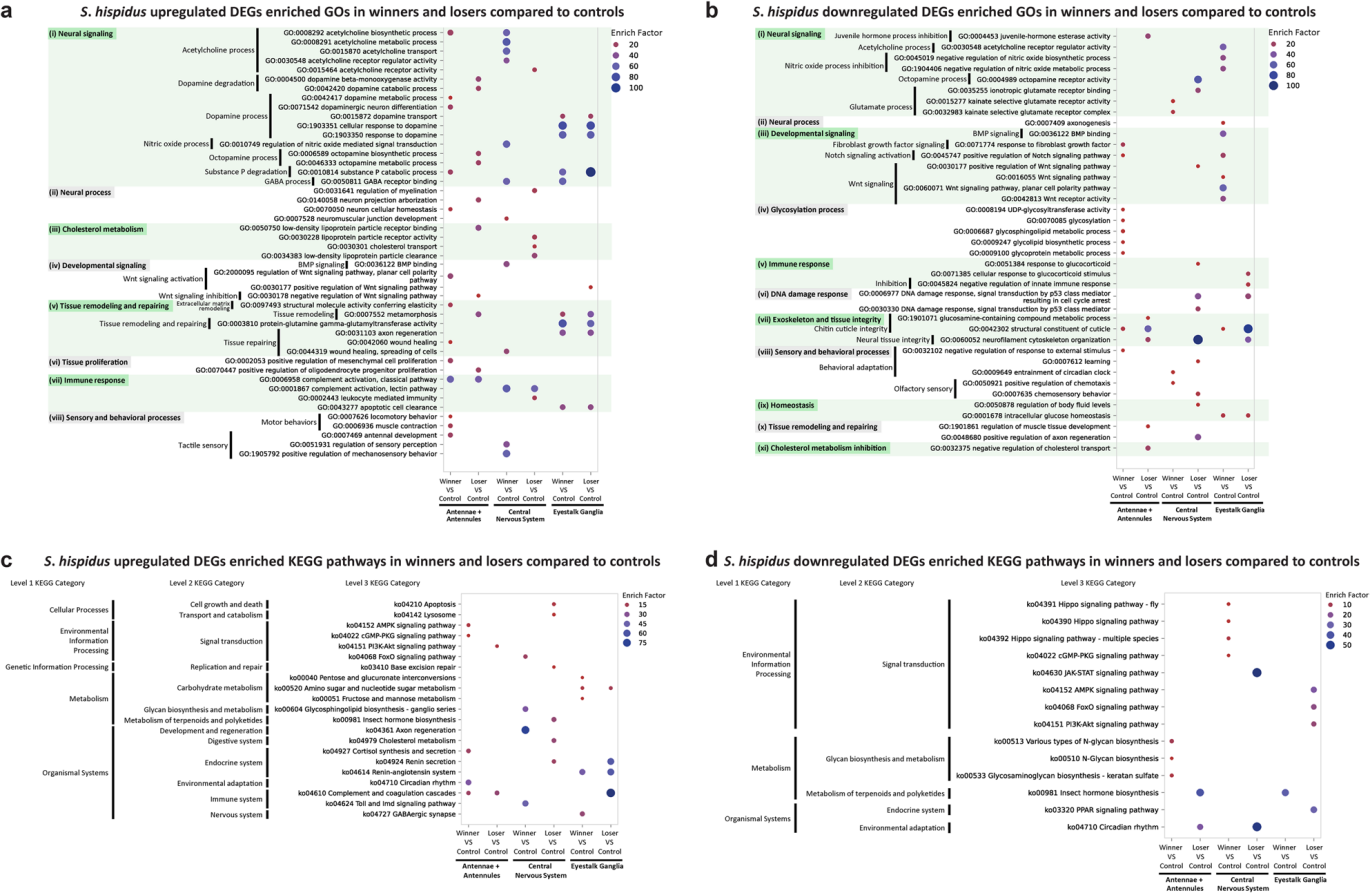
**a** Upregulated DEGs enriched GOs, (**b**) downregulated DEGs enriched GOs, (**c**) upregulated DEGs enriched KEGG pathways, and (**d**) downregulated DEGs enriched KEGG pathways in the three target organs (i.e., antennae + antennules, central nervous system, and eyestalk ganglia) of winners compared to controls and losers compared to controls in *S. hispidus*. All enriched GOs and KEGG pathways are statistically significant at *p* < 0.05. GO terms were grouped according to their typical functions, while KEGG pathways were by their standard three-level categorization. Figure 2 (**a**), (**b**), (**c**), and (**d**) are also provided individually in additional information file as Fig. S5

Despite some similarities in the GO categories and KEGG pathways enriched in the three organs (i.e., antennae + antennules, CNS, and eyestalk ganglia) shared between winners and losers of *S. hispidus* (e.g., sensory and behavioral processes as well as some immune and endocrine pathways), the GO categories and KEGG pathways were generally different (Fig. 2). Specifically, in the winners of *S. hispidus*, upregulated DEGs typically enriched GO categories for neural signaling (e.g., acetylcholine and dopamine processes) (Fig. 2a (i)), tissue remodeling and repairing (e.g., extracellular matrix) (Fig. 2a (v)), and sensory and behavioral processes (Fig. 2a (viii)), in addition to KEGG pathways involved in five organismal systems (i.e., nervous, immune, environmental adaptation, endocrine, and development and regeneration), carbohydrate metabolism, and signal transductions (e.g., ko04022 cGMP-PKG and ko04068 FoxO) (Fig. 2c). In contrast, downregulated DEGs of winners generally enriched GOs for developmental signaling (e.g., BMP, Notch, and Wnt) (Fig. 2b (iii)), as well as KEGG pathways for another set of signal transduction cascades (e.g., ko04390 Hippo), and glycan biosynthesis and metabolism (e.g., ko00510 N-glycan biosynthesis and ko00513 various types of N-glycan biosynthesis) (Fig. 2d).

In the losers of *S. hispidus*, upregulated DEGs also enriched GOs for neural signaling as in winners (but more on dopamine degradation and octopamine process for losers) (Fig. 2a (i)), along with KEGG pathways related to cellular processes (i.e., ko04210 apoptosis and ko04142 lysosome) and organismal systems, such as digestive (i.e., ko04979 cholesterol metabolism) and endocrine (e.g., ko04924 renin secretion) (Fig. 2c). However, unlike winners, downregulated DEGs in losers generally enriched a different group of GO categories and KEGG pathways. For example, GOs associated with DNA damage response (Fig. 2b (vi)), exoskeleton and tissue integrity (Fig. 2b (vii)), as well as sensory and behavioral processes (e.g., learning (GO:0007612); Fig. 2b (viii)), in addition to KEGG pathways related to environmental adaptation (i.e., ko04710 circadian rhythm), metabolism of cofactors and vitamins (i.e., ko00670 one-carbon pool by folate), and signal transductions (e.g., ko04068 FoxO and ko04630 JAK-STAT), were enriched (Fig. 2d).

**Fig. 3:**
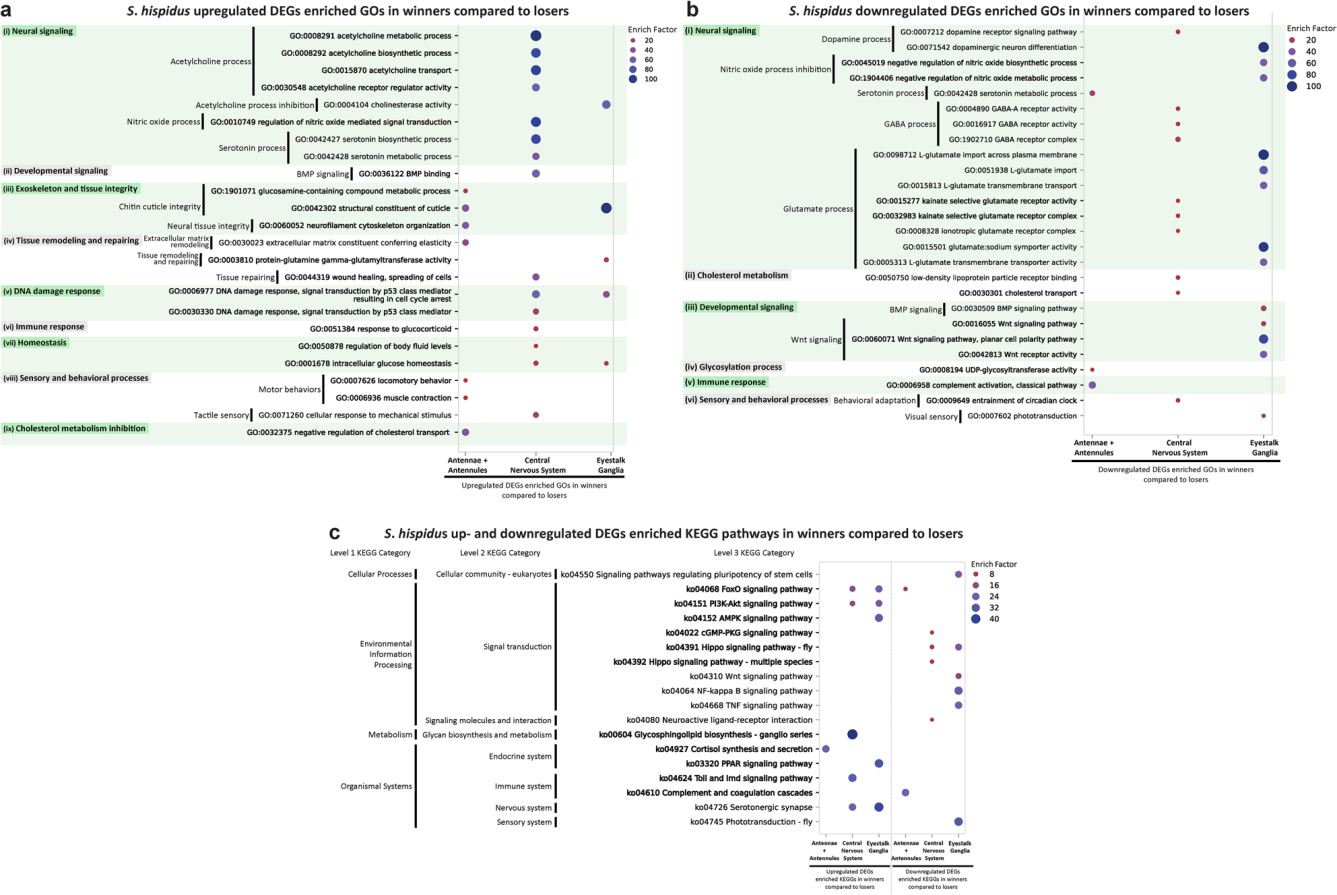
Up- and downregulated DEGs enriched (**a**, **b**) GOs and (**c**) KEGG pathways in the three target organs (i.e., antennae + antennules, central nervous system, and eyestalk ganglia) in winners compared to losers of *S. hispidus*. All enriched GOs and KEGG pathways are statistically significant at *p* < 0.05. GO terms were grouped according to their typical functions, while KEGG pathways were by their standard three-level categorization. Individually bolded GOs and KEGG pathways indicate that their DEGs were statistically significant in at least one treatment vs. control comparison. Figure 3 (**a**), (**b**), and (**c**) are also provided individually in additional information file as Fig. S6

Twelve of the GOs and three of the KEGG pathways were mutually enriched in the same organ of both winners and losers of *S. hispidus* (Table 2, 3 and 4). They generally belonged to categories such as neural signaling (i.e., substance P degradation and dopamine process; Table 2), endocrine system (i.e., ko04614 renin-angiotensin system; Table 3), exoskeleton and tissue integrity (i.e., structural constituent of cuticle (GO:0042302); Table 4), homeostasis (i.e., intracellular glucose homeostasis (GO:0001678); Table 4), as well as immune response and system (e.g., complement activation, classical pathway (GO:0006958); Table 2, and ko04610 complement and coagulation cascades; Table 3). Comparing winners with losers, winners showed a weaker upregulation of most DEGs that enriched for complement activation, classical pathway (GO:0006958) (Fig. 3b (v)), and ko04610 complement and coagulation cascades (Fig. 3c), whereas they exhibited a weaker downregulation of those that enriched for structural constituent of cuticle (GO:0042302) (Fig. 3a (iii)).

**Table 2:** Significantly enriched mutual GOs from upregulated DEGs in the same organ of both winners and losers compared to control individuals of *S. hispidus*

GO category	GO subcategory	GO terms and ID	Ontology	Enrichment factor of winner and loser	Organ of enrichment
Neural signaling	Substance P degradation	Substance P catabolic process (GO:0010814)	BP	Winner: 55.79, Loser: 111.57	EG
	Dopamine process	Dopamine transport (GO:0015872)	BP	Winner: 20.29, Loser: 20.29	EG
	Dopamine process	Response to dopamine (GO:1903350)	BP	Winner: 66.94, Loser: 66.94	EG
	Dopamine process	Cellular response to dopamine (GO:1903351)	BP	Winner: 83.68, Loser: 83.68	EG
Tissue remodeling and repairing	Tissue remodeling and repairing	Protein-glutamine gamma-glutamyltransferase activity (GO:0003810)	MF	Winner: 75.22, Loser: 60.17	EG
	Tissue remodeling	Metamorphosis (GO:0007552)	BP	Winner: 22.31, Loser: 44.63	EG
	Tissue repairing	Axon regeneration (GO:0031103)	BP	Winner: 39.38, Loser: 39.38	EG
Immune response		Complement activation, lectin pathway (GO:0001867)	BP	Winner: 67.95, Loser: 56.17	CNS
		Complement activation, classical pathway (GO:0006958)	BP	Winner: 54.14, Loser: 49.05	AA
		Apoptotic cell clearance (GO:0043277)	BP	Winner: 30.43, Loser: 30.43	EG

**Table 3:** Significantly enriched mutual KEGG pathways from upregulated DEGs in the same organ of both winners and losers compared to control individuals of *S. hispidus*

Level 1 KEGG category	Level 2 KEGG category	KO ID and pathway	Enrichment factor of winner and loser	Organ of enrichment
Organismal Systems	Endocrine system	ko04614 Renin-angiotensin system	Winner: 41.20, Loser: 56.18	EG
	Immune system	ko04610 Complement and coagulation cascades	Winner: 18.19, Loser: 22.33	AA
Metabolism	Carbohydrate metabolism	ko00520 Amino sugar and nucleotide sugar metabolism	Winner: 9.21, Loser: 12.56	EG

**Table 4:** Significantly enriched mutual GOs from downregulated DEGs in the same organ of both winners and losers compared to control individuals of *S. hispidus*

GO category	GO subcategory	GO terms and ID	Ontology	Enrichment factor of winner and loser	Organ(s) of enrichment
Exoskeleton and tissue integrity	Chitin cuticle integrity	Structural constituent of cuticle (GO:0042302)	MF	Winner: 11.78, Loser: 50.83 for AA Winner: 7.78, Loser: 93.80 for EG	AA and EG
Homeostasis		Intracellular glucose homeostasis (GO:0001678)	BP	Winner: 12.00, Loser: 12.06	EG

All GOs and KEGG pathways shown in this table are statistically significant at *p* < 0.05. GO terms were grouped according to their typical functions within GO (sub-)categories, while KEGG pathways were by their standard three-level categorization

*AA* Antennae + Antennules, *BP* Biological Processes, *CNS* Central Nervous System, *EG* Eyestalk Ganglia, *MF* Molecular Function

### GO and KEGG enrichment analysis results of *S. cyanoscelis*

**Fig. 4:**
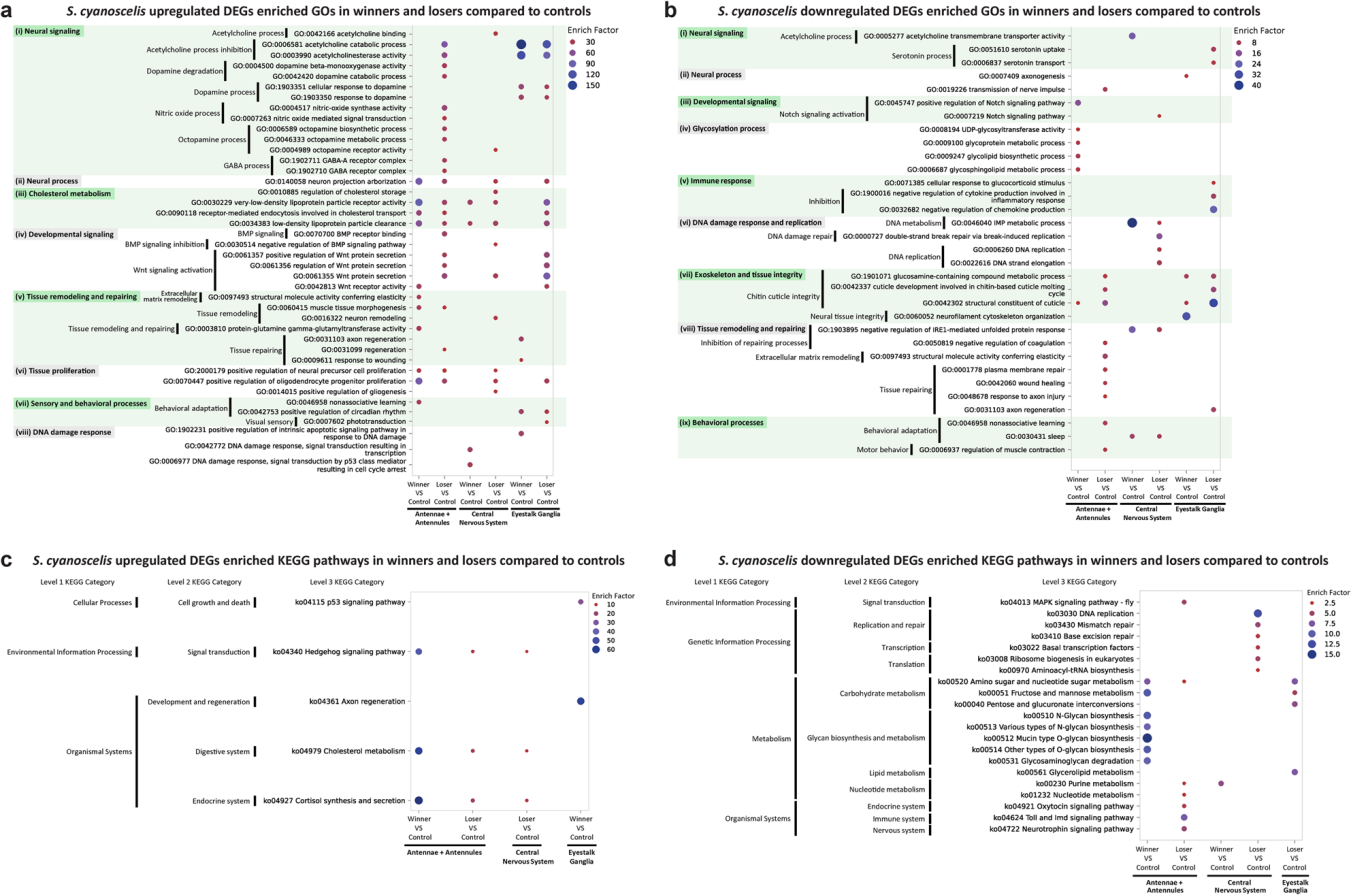
**a** Upregulated DEGs enriched GOs, (**b**) downregulated DEGs enriched GOs, (**c**) upregulated DEGs enriched KEGG pathways, and (d) downregulated DEGs enriched KEGG pathways in the three target organs (i.e., antennae + antennules, central nervous system, and eyestalk ganglia) of winners compared to controls and losers compared to controls in *S. cyanoscelis*. GO terms were grouped according to their typical functions, while KEGG pathways were by their standard three-level categorization. All enriched GOs and KEGG pathways are statistically significant at *p* < 0.05. Figure 4 (**a**), (**b**), (**c**), and (**d**) are also provided individually in additional information file as Fig. S7

Similar to *S. hispidus*, different sets of enriched GO categories and KEGG pathways were generally observed between winners and losers of *S. cyanoscelis* (Fig. 4). Specifically, in the winners of *S. cyanoscelis*, upregulated DEGs enriched GOs involved in neural signaling (e.g., dopamine process; Fig. 4a (i)), tissue remodeling and repairing (Fig. 4a (v)), sensory and behavioral processes (e.g., non-associative learning (GO:0046958); Fig. 4a (vii)), and DNA damage response (Fig. 4a (viii)), in addition to KEGG pathways that were mostly related to organismal systems (e.g., development and regeneration) and cellular processes (i.e., ko04115 p53 signaling pathway) (Fig. 4c). Conversely, downregulated DEGs in winners typically enriched GOs related to glycosylation process (Fig. 4b (iv)), along with three different sets of KEGG metabolic pathways, including carbohydrate (e.g., ko00051 fructose and mannose metabolism), glycan (e.g., ko00510 N-glycan biosynthesis and ko00513 various types of N-glycan biosynthesis), and nucleotide (i.e., ko00230 purine metabolism) (Fig. 4d).

In the losers of *S. cyanoscelis*, most upregulated DEGs enriched GOs for neural signaling (e.g., dopamine, nitric oxide, and octopamine; Fig. 4a (i)) and developmental signaling (e.g., BMP and Wnt; Fig. 4a (iv)), along with KEGG pathways related to signal transduction (i.e., ko04340 Hedgehog) as well as digestive (i.e., ko04979 cholesterol metabolism) and endocrine systems (i.e., ko04927 cortisol synthesis and secretion) (Fig. 4c). In contrast, downregulated DEGs in losers mainly enriched GOs for another set of neural signaling (e.g., serotonin process; Fig. 4b (i)), exoskeleton and tissue integrity (Fig. 4b (vii)), and tissue remodeling and repairing (e.g., wound healing (GO:0042060); Fig. 4b (viii)), in addition to KEGG pathways associated with genetic information processing (i.e., transcription, translation, and replication and repair), three organismal systems (i.e., endocrine, immune, and nervous), carbohydrate metabolism, and cofactor and vitamin metabolism (i.e., ko00670 one-carbon pool by folate) (Fig. 4d).

**Fig. 5:**
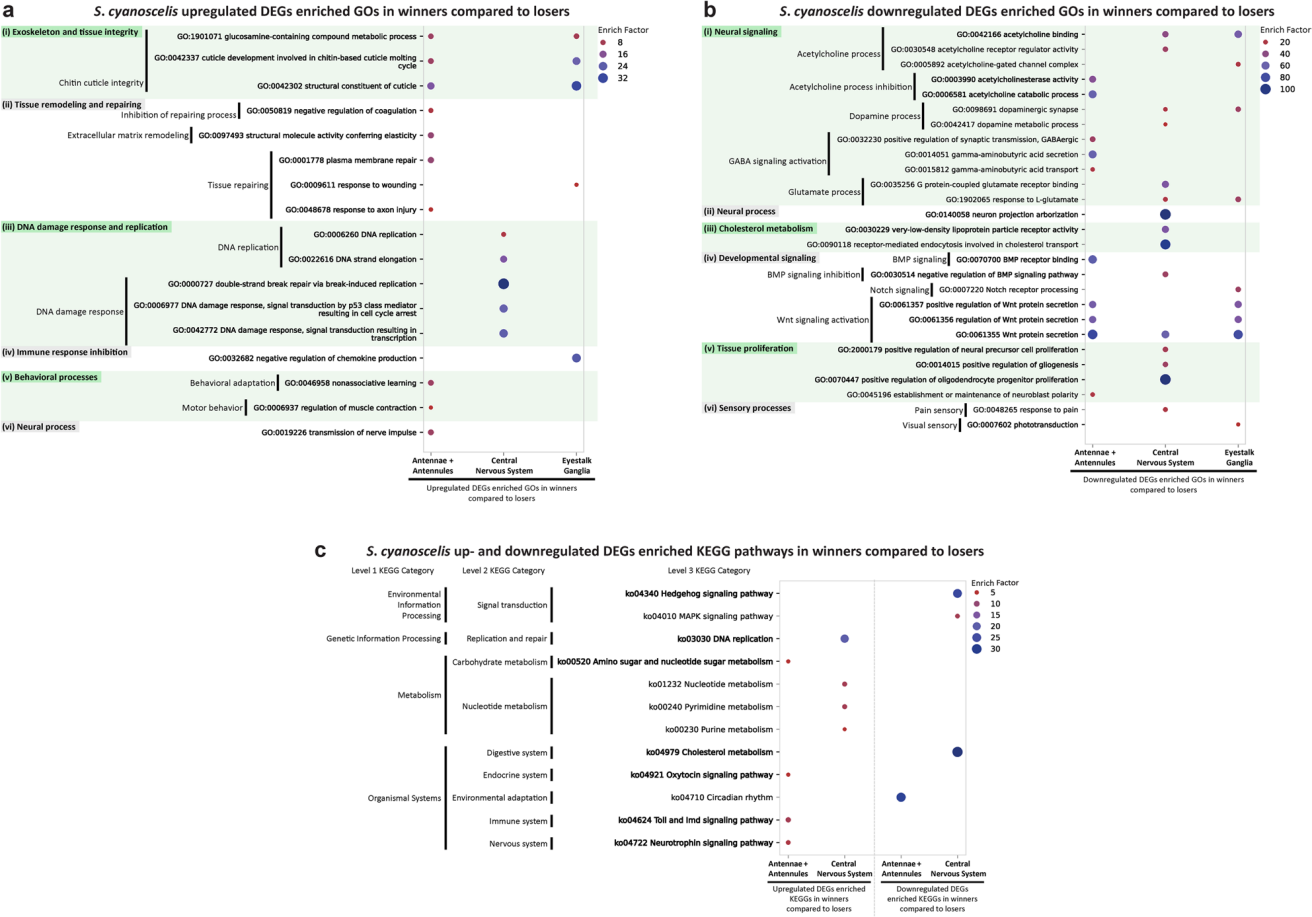
Up- and downregulated DEGs enriched (**a**, **b**) GOs and (**c**) KEGG pathways in the three target organs (i.e., antennae + antennules, central nervous system, and eyestalk ganglia) in winners compared to losers of *S. cyanoscelis*. All enriched GOs and KEGG pathways are statistically significant at *p* < 0.05. GO terms were grouped according to their typical functions, while KEGG pathways were by their standard three-level categorization. Individually bolded GOs and KEGG pathways indicate that their DEGs were statistically significant in at least one treatment vs. control comparison. Figure 5 (**a**), (**b**), and (**c**) are also provided individually in additional information file as Fig. S8

Seventeen and four of the GOs and KEGG pathways, respectively, were mutually enriched in the same organ of both winners and losers of *S. cyanoscelis* (Table 5, 6, 7 and 8). The seventeen GOs generally belonged to categories such as neural signaling (i.e., acetylcholine process inhibition and dopamine process; Table 5), cholesterol metabolism (e.g., very-low-density lipoprotein particle receptor activity (GO:0030229); Table 5), tissue proliferation (i.e., oligodendrocyte (GO:0070447) and neural precursor cell (GO:2000179); Table 5), exoskeleton and tissue integrity (i.e., structural constituent of cuticle (GO:0042302) and glucosamine-containing compound metabolic process (GO:1901071); Table 6), as well as behavioral processes (e.g., sleep (GO:0030431); Table 6). On the other hand, the four mutually enriched KEGG pathways belonged to endocrine and digestive systems, signal transduction, and carbohydrate metabolism (Tables 7 and 8). Comparing winners with losers, winners generally showed a weaker downregulation of DEGs that enriched for structural constituent of cuticle (GO:0042302) and glucosamine-containing compound metabolic process (GO:1901071) (Fig. 5a (i)).

**Table 5:** Significantly enriched mutual GOs from upregulated DEGs in the same organ of both winners and losers compared to control individuals of *S. cyanoscelis*

GO category	GO subcategory	GO terms and ID	Ontology	Enrichment factor of winner and loser	Organ(s) of enrichment
Neural signaling	Acetylcholine process inhibition	Acetylcholinesterase activity (GO:0003990)	MF	Winner: 131.23, Loser: 87.49	EG
	Acetylcholine process inhibition	Acetylcholine catabolic process (GO:0006581)	BP	Winner: 178.10, Loser: 118.73	EG
	Dopamine process	Response to dopamine (GO:1903350)	BP	Winner: 35.62, Loser: 23.75	EG
	Dopamine process	Cellular response to dopamine (GO:1903351)	BP	Winner: 41.56, Loser: 27.70	EG
Neural process		Neuron projection arborization (GO:0140058)	BP	Winner: 82.92, Loser: 35.03	AA
Cholesterol metabolism		Very-low-density lipoprotein particle receptor activity (GO:0030229)	MF	Winner: 93.29, Loser: 39.41 for AA; Winner: 35.71, Loser: 25.26 for CNS	AA, CNS
		Low-density lipoprotein particle clearance (GO:0034383)	BP	Winner: 65.85, Loser: 27.82 for AA Winner: 25.21, Loser: 35.65 for CNS	AA, CNS
		Receptor-mediated endocytosis involved in cholesterol transport (GO:0090118)	BP	Winner: 46.64, Loser: 19.70	AA
Tissue remodeling and repairing	Tissue remodeling	Muscle tissue morphogenesis (GO:0060415)	BP	Winner: 27.30, Loser: 11.53	AA
Tissue proliferation		Positive regulation of oligodendrocyte progenitor proliferation (GO:0070447)	BP	Winner: 82.92, Loser: 35.03	AA
		Positive regulation of neural precursor cell proliferation (GO:2000179)	BP	Winner: 21.74, Loser: 13.77	AA
Sensory and behavioral processes	Behavioral adaptation	Positive regulation of circadian rhythm (GO:0042753)	BP	Winner: 32.81, Loser: 21.87	EG

**Table 6:** Significantly enriched mutual GOs from downregulated DEGs in the same organ of both winners and losers compared to control individuals of *S. cyanoscelis*

GO category	GO subcategory	GO terms and ID	Ontology	Enrichment factor of winner and loser	Organ(s) of enrichment
DNA damage response and replication	DNA metabolism	IMP metabolic process (GO:0046040)	BP	Winner: 44.51, Loser: 4.88	CNS
Exoskeleton and tissue integrity	Chitin cuticle integrity	Structural constituent of cuticle (GO:0042302)	MF	Winner: 4.29, Loser: 14.09 for AA Winner: 5.53, Loser: 35.04 for EG	AA and EG
	Chitin cuticle integrity	Glucosamine-containing compound metabolic process (GO:1901071)	BP	Winner: 7.59, Loser: 10.52	EG
Tissue remodeling and repairing	Inhibition of repairing processes	Negative regulation of IRE1-mediated unfolded protein response (GO:1903895)	BP	Winner: 19.91, Loser: 8.74	CNS
Behavioral processes	Behavioral adaptation	Sleep (GO:0030431)	BP	Winner: 9.23, Loser: 7.08	CNS

**Table 7:** Significantly enriched mutual KEGG pathways from upregulated DEGs in the same organ of both winners and losers compared to control individuals of *S. cyanoscelis*

Level 1 KEGG category	Level 2 KEGG category	KO ID and pathway	Enrichment factor of winner and loser	Organ of enrichment
Organismal Systems	Endocrine system	ko04927 Cortisol synthesis and secretion	Winner: 66.69, Loser: 17.15	AA
	Digestive system	ko04979 Cholesterol metabolism	Winner: 57.16, Loser: 14.70	AA
Environmental Information Processing	Signal transduction	ko04340 Hedgehog signaling pathway	Winner: 44.46, Loser: 11.43	AA

**Table 8:** Significantly enriched mutual KEGG pathways from downregulated DEGs in the same organ of both winners and losers compared to control individuals of *S. cyanoscelis*

Level 1 KEGG category	Level 2 KEGG category	KO ID and pathway	Enrichment factor of winner and loser	Organ of enrichment
Metabolism	Carbohydrate metabolism	ko00520 Amino sugar and nucleotide sugar metabolism	Winner: 7.67, Loser: 2.82	AA

All GOs and KEGG pathways shown in this table are statistically significant at *p* < 0.05. GO terms were grouped according to their typical functions within GO (sub-)categories, while KEGG pathways were by their standard three-level categorization

*AA* Antennae + Antennules, *BP* Biological Processes, *CNS* Central Nervous System, *EG* Eyestalk Ganglia, *MF* Molecular Function

### Mutually enriched GOs and KEGG pathways in *S. hispidus* and *S. cyanoscelis*

**Fig. 6:**
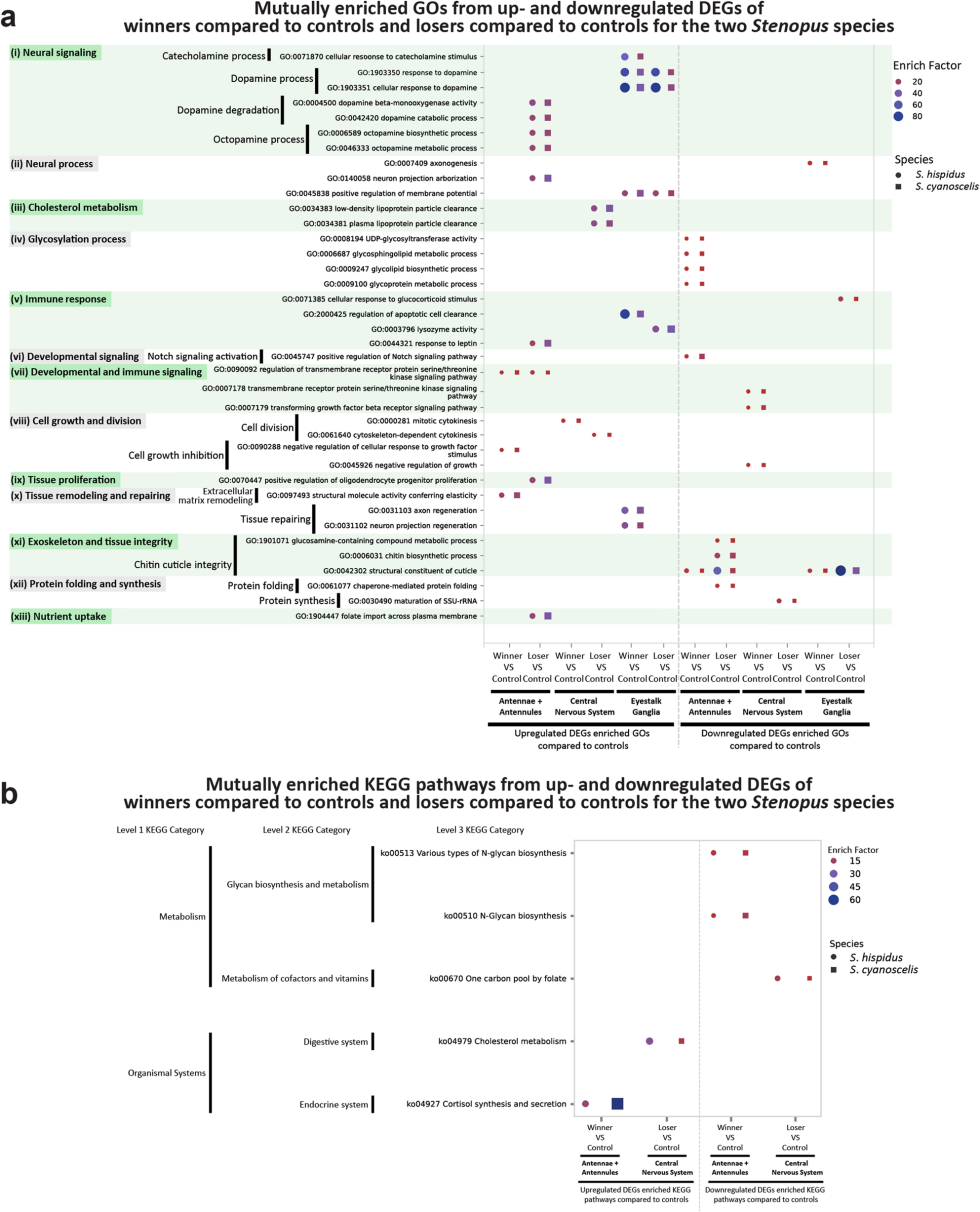
Mutually enriched (**a**) GOs and (**b**) KEGG pathways from upregulated and downregulated DEGs in the same organ (i.e., antennae + antennules, central nervous system, and eyestalk ganglia) of winners compared to control individuals and losers compared to control individuals of both *Stenopus* species. All enriched GOs and KEGG pathways are statistically significant at *p* < 0.05. GO terms were grouped according to their typical functions, while KEGG pathways were by their standard three-level categorization. Figure 6 (**a**) and (**b**) are also provided individually in additional information file as Fig. S9

Thirty-eight GOs and five KEGG pathways were mutually enriched in the same organ of winners or losers in the two studied *Stenopus* species, with winners and losers typically enriching different GOs and KEGG pathways (Fig. 6). Generally, mutually up- and downregulated DEGs of winner *Stenopus* enriched for GOs categorized under neural signaling (e.g., dopamine process; Fig. 6a (i)), cell growth and division (Fig. 6a (viii)), and tissue remodeling and repairing (Fig. 6a (x)), while the enriched KEGG pathways were associated with endocrine system (i.e., ko04927 cortisol synthesis and secretion) and glycan biosynthesis and metabolism (i.e., ko00510 N-glycan biosynthesis and ko00513 various types of N-glycan biosynthesis) (Fig. 6b). On the other hand, DEGs of loser *Stenopus* generally enriched for GO categories such as neural signaling (but more on dopamine degradation and octopamine process; Fig. 6a (i)), exoskeleton and tissue integrity (Fig. 6a (xi)), and protein folding and synthesis (Fig. 6a (xii)), in addition to KEGG pathways related to digestive system (i.e., ko04979 cholesterol metabolism) and metabolism of cofactors and vitamins (i.e., ko00670 one-carbon pool by folate) (Fig. 6b).

Among the 38 GOs, five of them, including response to dopamine (GO:1903350) (Fig. 6a (i)), cellular response to dopamine (GO:1903351) (Fig. 6a (i)), positive regulation of membrane potential (GO:0045838) (Fig. 6a (ii)), regulation of transmembrane receptor protein serine/threonine kinase signaling pathway (GO:0090092) (Fig. 6a (vii)), and structural constituent of cuticle (GO:0042302) (Fig. 6a (xi)), were mutually enriched in the same organ of both winners and losers of both *Stenopus* species, suggesting their importance to both dominants and subordinates in this genus. Comparing winners with losers, winners of both *Stenopus* species generally showed a weaker downregulation of DEGs that enriched for structural constituent of cuticle (GO:0042302) (additional information file Fig. S10 (i)).

### Common pattern of neural signaling systems-related DEGs expressed or enriched in *Stenopus* after fighting

Twelve aggression-related neural signaling systems were identified in the two studied *Stenopus* species (additional information file Table S5), nine of which were enriched at the gene set level (i.e., identified through GO and KEGG results), and the remaining three were found at the gene level (i.e., neurohormone-related DEGs that lacked GO and KEGG annotations). To determine whether the level of each neural signaling system was elevated or reduced in each organ for each pairwise comparison, the results of the differential gene expression analyses and the functions of key DEGs associated with each system, such as receptors, transporters, and genes involved in their synthesis and degradation (additional information file Table S2), were manually examined. While genes related to these signaling systems, such as serotonin and crustacean hyperglycemic hormones, were often differentially expressed or enriched in a species-specific manner or in different organs in the two studied species (additional information file Table S5), the levels of four of them, including dopamine, acetylcholine, octopamine, and glutamate, were mutually elevated or reduced in the same organ of both species (Table 9). Specifically, dopamine levels were enhanced in the eyestalk ganglia of both winners and losers but were reduced in the antennae + antennules of losers. Acetylcholine level was reduced in the eyestalk ganglia of winners but were elevated in the CNS of losers, while octopamine level was elevated in the antennae + antennules of losers. As for glutamate, although they were not significantly enriched in either winners or losers when compared to control individuals, their levels were significantly lower in the CNS of winners than in losers.

**Table 9:** Common pattern of neural signaling systems-related DEGs potentially mediating agonistic behaviors in *Stenopus*, identified in the same organ across the three pairwise comparisons in the two studied species

		Winner VS Control			Loser VS Control			Winner VS Loser		
Neural signaling systems		Antennae + Antennules	Central nervous system	Eyestalk ganglia	Antennae + Antennules	Central nervous system	Eyestalk ganglia	Antennae + Antennules	Central nervous system	Eyestalk ganglia
^a^Gene set level	Dopamine			$\:+$	$\:-$		$\:+$			
	Acetylcholine			$\:-$		$\:+$				$\:-$
	Octopamine				$\:+$					
	Glutamate								$\:-$	

^a^Gene set level refers to the neural signaling systems-related DEGs identified through GO and KEGG enrichment results.$+$, level elevated.$-$, level reduced

## Discussion

### Potentially major neural signaling systems involved in mediating fighting interactions in *Stenopus*

Our differential gene expression analysis as well as GO and KEGG enrichment analyses revealed that, among the twelve neural signaling systems differentially expressed or enriched after fighting in *Stenopus*, DEGs of only four of these systems, including dopamine, acetylcholine, octopamine, and glutamate, showed conserved changes in levels within the same organ between the two studied species. These four systems are therefore considered potentially major ones in mediating agonistic behaviors and fighting interactions in *Stenopus*. Among these four systems, we believe that dopamine and acetylcholine might be relatively more important than the other two. This hypothesis is based on the fact that the changes of octopamine gene expression level in antennae + antennules were not directly involved in regulating agonism but were more associated with sensory detection according to previous studies [64, 65] (see the paragraph after the next for explanation), while DEGs associated with glutamate signaling system were not significantly enriched in either winners or losers when compared to control individuals, despite being significantly enriched in winners compared to losers (Table 9).

Dopamine expression levels were consistently elevated in the eyestalk ganglia of both winners and losers in the two *Stenopus* species. Numerous studies on both vertebrate and invertebrate models have shown that dopamine is a crucial neural signaling molecule controlling agonistic behaviors. In invertebrates, for instance, Alekseyenko et al. [66] demonstrated that activating dopaminergic neurons resulted in increased aggression in fruit flies, while Bubak et al. [67] found that only the increase of dopamine, but not that of other neural signaling molecules (e.g., serotonin or octopamine), was associated with physical fights in ants. Thus, the increased dopamine expression levels in the eyestalk ganglia of both winners and losers of *Stenopus* might correlate with the physical fighting interactions between the two contestants and the associated changes of agonism compared to control individuals. However, as dopamine expression levels in the eyestalk ganglia did not differ significantly between winners and losers, we believe that their behavioral and motivational differences might not depend solely on dopamine.

In addition to the elevation in the eyestalk ganglia, dopamine gene expression was also found to be reduced in the antennae + antennules of losers in both *Stenopus* species, which correlated with the elevation of octopamine expression. The functions of octopamine and dopamine in these two peripheral sensory organs of crustaceans are typically associated with modulating the detection of various sensory cues, such as olfactory, pheromonal, and gustatory, for social communication and recognition [65, 68, 69], which appeared to be indirectly related to the regulation of aggression. Therefore, we believe that the alterations of dopamine and octopamine expression in the antennae + antennules of losers might be more associated with detecting the presence or location of winners rather than directly mediating agonistic behaviors or physical fights in *Stenopus*.

Furthermore, we observed that the levels of DEGs associated with acetylcholine signaling system were lower in the eyestalk ganglia of winners but higher in the CNS of losers. Numerous studies have illustrated that acetylcholine is critical for controlling agonistic interactions in animals [9, 70]. For example, vertebrate and invertebrate studies have both demonstrated that a reduction of acetylcholine level generally leads to an increase of aggression [14, 71, 72]. These findings align with our results in *Stenopus*, where the decrease of acetylcholine expression in the eyestalk ganglia of winners might be associated with increased aggression and the exhibition of more frequent and/or prolonged winner-specific aggressive behaviors, including threatening display and rushing [31]. Conversely, the elevated acetylcholine expression in the CNS of losers might correlate with their more frequent and/or prolonged loser-specific appeasement behaviors, including crouching and back-swimming [31], as acetylcholine in the CNS has been associated with the formation of aversive memories and stress in many animals, including insects [73–75]. In particular, our results resembled Mineur et al. (2013)’s findings in mice, which showed that anxiety and depressive behaviors exhibited by losers following repeated social defeat were due to increased acetylcholine level in their CNS [76]. Similarly, Kinoshita and Okamoto [70] found that acetylcholine activation in the CNS of zebrafish losers was responsible for the switch from aggressive to submissive behaviors upon the establishment of a dominant-subordinate relationship to avoid further injuries. Collectively, these studies suggested that the changes of acetylcholine expression levels might correlate with the behavioral and motivational differences observed between winners and losers in *Stenopus*. Interestingly, many vertebrate and invertebrate studies have also demonstrated that acetylcholine occasionally mediates, and in some cases, reciprocally influences or modulates the release of dopamine [77, 78]. The interplay of these two signaling molecules has also been found to be important for fine-tuning synaptic transmission, modulating animal behaviors and motivation, and maintaining proper physiological functions (e.g., neural processing and cognition) [79–81]. However, whether the changes of both acetylcholine and dopamine expression levels in the eyestalk ganglia of winners might be the result of their interplay or co-signaling of agonistic behaviors and fighting interactions in *Stenopus* remains to be investigated. Additionally, the relatively higher glutamate expression level in the CNS of losers compared to winners might also be related to the exhibition of appeasement behaviors upon the establishment of dominance, which was generally consistent with the findings of Kinoshita and Okamoto [70], who reported enhanced glutamatergic signaling in the CNS of zebrafish losers.

While the present study has provided important insights into the potentially major neural signaling systems correlated with agonistic behaviors and fighting interactions in *Stenopus*, substantial future work, such as genetic and hormonal manipulation experiments (which are common in studies of insects and other crustaceans, respectively), is needed to validate the present results and to understand the functions of these systems in aggression. For example, an interventional injection experiment to increase dopamine and deplete acetylcholine biological levels in the eyestalk ganglia of the shrimps could be performed to observe whether more frequent and/or prolonged winner-specific aggressive behaviors are elicited. In contrast, injecting acetylcholine into the CNS and dopamine into the eyestalk ganglia should yield highly sensitive individuals that exhibit more frequent and/or prolonged loser-specific appeasement behaviors. Moreover, metabolomic investigation is essential for a comprehensive understanding of the complex regulation of *Stenopus* agonistic behaviors at different molecular levels, and is a prerequisite for injecting and depleting appropriate biological amounts of dopamine and acetylcholine in the aforementioned injection experiment [82, 83]. Furthermore, investigations into the functional roles of different (sub-)types of receptors associated with the identified potentially major neural signaling systems are critical for fully understanding their mediation and downstream signaling cascades, as the functions of receptors may vary in terms of their cellular localization, which could ultimately affect animals’ aggressiveness [84, 85]. Besides, quantitative real-time PCR (qRT-PCR) validation in the future will be advantageous for confirming the DEG results of this study. Additionally, although this study focused on examining the genetic and neural signaling basis and physiological pathways potentially mediating agonistic behaviors in female *Stenopus*, which are believed to be generally applicable to males, we do not disregard the possibility of some slight variations in the use of neural signaling systems between females and males. Therefore, obtaining transcriptomic data from males in the future for comparisons will be beneficial to supplement the females’ data and further improve our understanding of the genetic and neural signaling basis and pathways mediating agonistic behaviors in *Stenopus*.

### Comparison of aggression-related neural signaling systems between *Stenopus* and other pan-crustaceans

For the five insect groups commonly studied for aggression (i.e., hymenopterans, orthopterans, coleopterans, blattodeans, and dipterans), at least 11 common neural signaling systems associated with mediating agonistic behaviors (in the absence of environmental and anthropogenic stressors) have been identified (Table 10) [9, 11, 23, 86]. Among these 11 systems, dopamine and octopamine serve as the major ones, as they are present in all five insect groups examined. They are closely followed by serotonin, which is documented in four groups (including hymenopterans, orthopterans, coleopterans, and dipterans), and juvenile hormone, which is identified in three groups (including hymenopterans, coleopterans, and blattodeans). The remaining aggression-related neural signaling systems are more taxon-specific and are involved in only one or two groups. These findings suggest that their roles in mediating aggression in insects are conserved to a certain degree and variation between groups exists. For crustaceans, a similar group of major neural signaling systems, including serotonin, dopamine, and octopamine, is observed (Table 10). Among them, serotonin is the most conserved owing to its presence in all fifteen agonistic genera from five crustacean taxa examined (including stomatopods, carideans, astacideans, anomurans, and brachyurans). However, certain systems appear to be specific to insects and crustaceans, including tachykinin, juvenile hormone, nitric oxide, neuropeptide F, and cholecystokinin-like peptide utilized by the former, while SIFamide and crustacean hyperglycemic hormone by the latter. Similar functional variation also seems to exist at a lower taxonomic level within crustaceans; for example, crustacean hyperglycemic hormone is only involved in four out of fifteen genera.

**Table 10:** Common aggression-related neural signaling systems documented in the examined pan-crustaceans, summarized from studies conducted in the absence of environmental and anthropogenic stressors

				Neural signaling systems											
Taxonomic group			DA	5-HT	OA	Substance P/ TK	ACh	JH/ MF	NO	Glutamate	GABA	NPF	CCK-like	SIFamide	CHH
Insects	Hymenopterans		x	x	x	x	x	x		x	x				
	Orthopterans		x	x	x				x						
	Coleopterans		x	x	x			x							
	Blattodeans		x		x			x							
	Dipterans		x	x	x	x	x			x	x	x	x		
Crustaceans	Stomatopods	*Neogonodactylus*	x	x											
		*Gonodactylus*	x	x											
		*Haptosquilla*	x	x	x										
	Carideans	*Macrobrachium*	x	x	x					x				x	
	Astacideans	*Homarus*		x	x										
		*Procambarus*	x	x	x		x				x				x
		*Faxonius*	x	x											
		*Astacus*		x											
	Anomurans	*Munida*		x	x										
		*Pagurus*	x	x											
	Brachyurans	*Eriocheir*	x	x											x
		*Portunus*	x	x											x
		*Scylla*	x	x											x
		*Chasmagnathus*		x	x										
		*Carcinus*	x	x	x						x				

*DA* Dopamine, *5-HT* Serotonin, *OA* Octopamine, *TK* Tachykinin (homolog of substance P in insects), *ACh* Acetylcholine, *JH* Juvenile hormone, *MF* Methyl farnesoate (homolog of juvenile hormone in crustaceans), *NO* Nitric oxide, *GABA* Gamma-aminobutyric acid, *NPF* Neuropeptide F, *CCK-like* Cholecystokinin-like peptide, *CHH* Crustacean hyperglycemic hormone. x, has potential neural signaling roles in that group. References from [9, 14–16, 20, 22, 23, 26, 27, 67, 87–110].

Compared to the examined pan-crustaceans (Table 10), *Stenopus* appears to stand out for seemingly utilizing a relatively different set of neural signaling systems, primarily dopamine and acetylcholine over serotonin (Table 9), for potentially mediating its agonistic behaviors and fighting interactions. While serotonin system has been found to be potentially involved in mediating agonistic behaviors in all fifteen crustacean genera and almost all insect groups examined in Table 10, genes related to this system were not differentially expressed in any of the organs of either winners or losers when compared to control individuals, except for the eyestalk ganglia of losers in *S. cyanoscelis*. This seems to suggest that serotoninergic system might not be as essential as other neural signaling systems, such as dopamine and acetylcholine, in potentially mediating agonistic interactions in *Stenopus*. However, given that serotonin is widely conserved among pan-crustaceans with potential aggression-signaling functions, further studies are clearly needed to clarify its role in mediating aggression in this genus.

Among the documented neural signaling systems potentially involved in mediating aggression in crustaceans (Table 10), the roles and significance of some, such as serotonin and octopamine in carideans [87] as well as in certain anomurans [88] and astacideans [89], were concluded solely based on behavioral changes following hormonal injections (or their chemical analogs). However, these conclusions lacked prior confirmation of the differential expression of neural signaling systems-related genes through methods such as RNA-sequencing as performed in this study, or of the presence of neural signaling molecules through methods such as gas chromatography/mass spectrometry as in Sneddon et al. [90], during or after fighting. As this anthropogenically induced behavioral change does not necessarily imply that these signaling systems or molecules are naturally utilized during their fights, to ensure their ecological relevance in crustaceans, it is recommended to conduct functional validation studies on specific targets only after confirming their presence or differential expression in response to fighting interactions.

### Sensory and behavioral process alterations after fighting in *Stenopus*

Our GO and KEGG enrichment results revealed that DEGs related to sensory functions were enriched in various organs of *Stenopus*, such as tactile sensory (e.g., GOs related to the regulation of mechanosensory behavior and response to mechanical stimulus) for winners, and visual sensory (e.g., GOs and KEGG pathways related to phototransduction) for losers. This suggests that winners and losers might rely on different sensory cues to detect their opponents after dominance was established, even though tactile cues were prerequisite for inducing fighting interactions in *Stenopus* [31]. The shift from the use of tactile cues to visual cues in losers after dominance was established might be attributed to the deprivation of their tactile sensory detection capabilities caused by the loss of their tactile sensory structure, e.g., cheliped(s) [111], as opposed to control individuals and winners whose bodies were relatively intact. Sensory compensation phenomena to (partially) counteract sensory disabilities or deficits are not unusual among crustaceans [111, 112]. Our results also corroborated those of Johnson (1969) [32], who discovered that other sensory cues, such as visual cues, were adopted to counteract the loss of tactile sensory detection capability in *S. hispidus*. These sensory compensations might be adaptive behaviors of *Stenopus*, aiding losers in locating surrounding winners and/or detecting potential threat signals to avoid further injuries. Notably, the enrichment of pain sensation in their CNS (i.e., response to pain (GO:0048265)) after injurious fights suggested that some *Stenopus* species might be capable of detecting pain, which is not unusual among arthropods, including decapod crustaceans [113, 114].

Furthermore, various behavioral processes-related GOs and KEGG pathways associated with learning (e.g., learning (GO:0007612) and non-associative learning (GO:0046958)) and regulation of circadian rhythm (e.g., entrainment of circadian clock (GO:0009649), positive regulation of circadian rhythm (GO:0042753), and ko04710 circadian rhythm) were generally altered in *Stenopus* winners and losers. These processes are known to be related to aggressiveness in other animals [115, 116]. For example, Hood and Amir concluded from various animal taxa that the exhibition of aggressive behaviors could disrupt normal rhythmic cycles of physiological activities [117]. Jiménez-Morales et al. showed that crayfish could learn and memorize their own social ranks and exhibit appropriate agonistic behaviors upon the establishment of social hierarchy [118]. The association of learning and circadian rhythm with fighting interactions in *Stenopus* suggests that a similar physiology and cognition of social hierarchy might also exist in this genus.

### Roles of metabolic and digestive pathways in fighting interactions of *Stenopus*

Two glycan biosynthesis and metabolic pathways (i.e., ko00510 N-glycan biosynthesis and ko00513 various types of N-glycan biosynthesis) were enriched in the antennae + antennules of winner *Stenopus*, while one digestive pathway (i.e., ko04979 cholesterol metabolism) and one cofactor and vitamin metabolic pathway (i.e., ko00670 one-carbon pool by folate) were enriched in the CNS of loser *Stenopus*. These pathways are generally associated with tissue or organ damage and repair, suggesting that fighting interactions in *Stenopus* are injurious to both contestants, as in most animal contests [119]. However, the potential utilization of different repairing pathways between winners and losers suggests that they might have experienced different types of injuries.

In winner *Stenopus*, various N-glycan biosynthetic pathways were enriched in the antennae + antennules, which appeared primarily associated with epithelial tissue and extracellular matrix injury, and wound healing [120, 121]. The synthesis of these glycans and their attachments is known to play vital roles in regulating diverse intracellular and extracellular functions across organisms, such as providing structural support to the extracellular matrix, regulating growth and differentiation, facilitating protein folding, and activating immune responses in response to infections and tissue injuries [122, 123]. The crustacean peripheral sensory organs (i.e., antennae and antennules) are relatively long structures exposed to the external environment and are often damaged during fights [124]. This is especially true in *Stenopus*, where fragmentation of these two structures during fighting seems to be more commonly observed compared to other crustacean aggression models, such as crayfish and lobsters [31]. These injuries may increase susceptibility to potential infections, necessitating the activation of immune responses and inflammation. Despite the vulnerability of antennae and antennules, they are capable of rapid growth and regeneration, typically resulting in a complete replica of the original after the next molt [125]. Thus, the enrichment of various N-glycan biosynthetic pathways in winner *Stenopus* suggests their potential pleiotropic roles in pathogen clearance, wound repair, and the regeneration of these vital but damage-prone organs.

Although damage to the antennae and antennules was also observed in the losers of the two studied *Stenopus* species, the lack of mutually enriched pathways suggests an inter-specific difference, which might depend on the situation and severity of injuries [126]. For instance, while complement and coagulation cascade, primarily associated with immune defense and wound healing [127, 128], was enriched in the antennae + antennules of losers in *S. hispidus*, another immune pathway, namely, Toll and Imd signaling, along with neurotrophin signaling that is typically associated with neural repair [129], were enriched in *S. cyanoscelis*. On the other hand, the mutually enriched cholesterol metabolism and one-carbon pool by folate pathways in the CNS of loser *Stenopus* were typically related to neural injuries and repair. While the former pathway is critical for neuron myelination and neuroprotection [130], the latter is vital for antioxidant defense and DNA repair for the proper functioning of the CNS [131–133]. Therefore, their enrichments in *Stenopus* fighting interactions suggested that these fights might also be associated with neural injuries, in addition to the epithelial wounds observed in winner *Stenopus*, which is not unusual in animal contests [134, 135]. Yet, as damage to the CNS could severely impact an individual’s motor coordination, and because the capacity for neural repair and regeneration following CNS damage in the adult stage of arthropods is relatively limited and often requires more time for repair compared to injuries in peripheral sensory organs and epithelia [119, 136, 137], the injuries in loser *Stenopus* seemed to be more traumatic than those in winners.

### Limitations

In this study, although we achieved a basic minimum sample size of three control individuals, three winners, and three losers for differential gene expression, as well as GO and KEGG enrichment analyses for both *Stenopus* species [138], we recognize that this sample size is relatively small due to challenges in purchasing these non-model crustaceans, which often lack a stable supply, especially during the COVID-19 pandemic; thus, some DEGs and neural signaling systems might have been missed. Future experiments with a larger sample size (e.g., six shrimps for each group) would be advantageous in reducing potential variations in gene expression and increasing the statistical power and accuracy of DEG, GO, and KEGG analyses [139, 140]. Yet, we believe that even with a larger sample size, the overall pattern of mutually expressed DEGs, enriched GOs and KEGG pathways, as well as the potentially major aggression-related neural signaling systems identified from the two studied *Stenopus* species, should remain generally consistent with the present results, given their consistent documentation despite the greater variations in gene expression caused by the smaller sample size in this study. Moreover, due to relatively low RNA concentrations in some organ samples, attributed to their small sizes in both *Stenopus* species (e.g., antennae + antennules), the RNA solutions from these samples were entirely used for RNA-sequencing, leaving no solutions available for subsequent quantitative real-time PCR (qRT-PCR) to verify the DEG results. While qRT-PCR validation would be beneficial, future functional and metabolomic validation of the current findings are prerequisites for more comprehensively understanding the neural signaling basis of agonistic behaviors in *Stenopus*.

## Conclusions

In conclusion, this study is the first that systematically examines the genetics of agonistic behaviors and fighting interactions of *Stenopus* shrimps. Our DEG along with GO and KEGG enrichment analyses suggested that *Stenopus* agonistic interactions might be systemic activities involving the simultaneous modulation and interplay of multiple signaling cascades, organismal systems, and metabolic pathways. In particular, winners and losers typically exhibited enriched GOs associated with neural signaling as well as sensory and behavioral processes. Regarding KEGG pathways, while those related to glycan biosynthesis and metabolism were enriched in winners, cholesterol metabolism and one-carbon pool by folate were enriched in losers. The different sets of pathways enriched in winners and losers suggested that while fighting interactions in *Stenopus* were injurious to both combatants, the damage in losers appeared to be more traumatic. Furthermore, while four neural signaling systems, including dopamine, acetylcholine, octopamine, and glutamate, were identified as potentially major mediators of agonistic behaviors and fighting interactions in both *Stenopus* species, the first two appeared to be relatively more important due to their potential roles in directly mediating agonism. However, subsequent functional and metabolomic studies are prerequisites for validating the present results. A comparison of the neural signaling systems involved in mediating aggression among pan-crustaceans suggested that *Stenopus* appeared to stand out by its seemingly major reliance on dopamine and acetylcholine, as opposed to the primarily serotonin-based regulation of aggression observed in all agonistic crustaceans and most insects examined in this study, such as fruit flies, lobsters, and crayfish models. This difference might make *Stenopus* a potentially valuable alternative model for studying behavioral genetics and aggression regulation aside from the traditional crustacean models.

## Supplementary Information

Additional file 1: Table S1 Time point of cheliped loss and the final endpoint for each fighting pair of S. *hispidus* and S. *cyanoscelis*. Table S2 Full list of annotated significantly up- and downregulated DEGs identified in *S. hispidus, S. cyanoscelis*, and mutually in *Stenopus*. Table S3 Full list of significantly enriched GOs from up- and downregulated DEGs identified in *S. hispidus, S. cyanoscelis*, and mutually in *Stenopus*. Table S4 Full list of significantly enriched KEGG pathways from up- and downregulated DEGs identified in *S. hispidus, S. cyanoscelis*, and mutually in *Stenopus*. Table S5 Neural signaling systems-related DEGs known to mediate agonism, identified in the three organs across the three pairwise comparisons of the two studied *Stenopus* species. Fig. S1 PCA plots for all expressed genes and DEGs only, for the three organs of winners, losers, and controls of *S. hispidus* and S. *cyanoscelis*. Fig. S2 Heatmaps and volcano diagrams for the three organs of winners and losers compared to controls for *S. hispidus* and *S. cyanoscelis*. Fig. S3 Heatmaps and volcano diagrams for the three organs of winners when compared to losers for *S. hispidus* and *S. cyanoscelis*. Fig. S4 Percentage of both up- and downregulated DEGs relative to the total number of DEGs in the three organs of the three comparisons of *S. hispidus* and *S. cyanoscelis*. Fig. S5 Individual components of Fig. 2 from the main text. Fig. S6 Individual components of Fig. 3 from the main text. Fig. S7 Individual components of Fig. 4 from the main text. Fig. S8 Individual components of Fig. 5 from the main text. Fig. S9 Individual components of Fig. 6 from the main text. Fig. S10 Mutually enriched GOs from upregulated and downregulated DEGs in the same organ of winners when compared to losers of both *Stenopus* species.

## Data Availability

All raw read data generated from *Stenopus hispidus* and *S. cyanoscelis* RNA samples have been deposited in NCBI Sequence Read Archive (SRA) under BioProject ID PRJNA1192195: https://www.ncbi.nlm.nih.gov/bioproject/?term=PRJNA1192195.

## Abbreviations

Antennae + antennules: Antennule and antennules combined
BGI: Beijing Genomics Institute
Bp: Base pair
BUSCO: Benchmarking Universal Single-Copy Orthologs
CNS: Central nervous system
DEGs: Differentially expressed genes
DNBSEQ: DNA nanoball sequencing
eggNOG: Evolutionary genealogy of genes: Non-supervised Orthologous Groups
GABA: Gamma-aminobutyric acid
Gb: Gigabyte
GO: Gene Ontology
KEGG: Kyoto Encyclopedia of Genes and Genomes
NCBI: National Center for Biotechnology Information
ORFs: Open reading frames
PCA: Principal component analysis
qRT-PCR: Quantitative real-time PCR
S. cyanoscelis: Stenopus cyanoscelis
S. hispidus: Stenopus hispidus
SRA: Short Read Archive
