# Supplementary material for: Transcriptomic investigation of agonistic behaviors of boxer shrimps (Stenopus species): insights into the potential neural signaling roles of dopamine and acetylcholine
Source: BMC Genomics. 2026 May 8;27:583. doi: 10.1186/s12864-026-12899-1 (PMC13330396; doi:10.1186/s12864-026-12899-1)
Supplement: Supplementary file 1 — Additional file 1: Table S1 Time point of cheliped loss and the final endpoint for each fighting pair of S. hispidus and S. cyanoscelis. Table S2 Full list of annotated significantly up- and downregulated DEGs identified in S. hispidus, S. cyanoscelis, and mutually in Stenopus. Table S3 Full list of significantly enriched GOs from up- and downregulated DEGs identified in S. hispidus, S. cyanoscelis, and mutually in Stenopus. Table S4 Full list of significantly enriched KEGG pathways from up- and downregulated DEGs identified in S. hispidus, S. cyanoscelis, and mutually in Stenopus. Table S5 Neural signaling systems-related DEGs known to mediate agonism, identified in the three organs across the three pairwise comparisons of the two studied Stenopus species. Fig. S1 PCA plots for all expressed genes and DEGs only, for the three organs of winners, losers, and controls of S. hispidus and S. cyanoscelis. Fig. S2 Heatmaps and volcano diagrams for the three organs of winners and losers compared to controls for S. hispidus and S. cyanoscelis. Fig. S3 Heatmaps and volcano diagrams for the three organs of winners when compared to losers for S. hispidus and S. cyanoscelis. Fig. S4 Percentage of both up- and downregulated DEGs relative to the total number of DEGs in the three organs of the three comparisons of S. hispidus and S. cyanoscelis. Fig. S5 Individual components of Fig. 2 from the main text. Fig. S6 Individual components of Fig. 3 from the main text. Fig. S7 Individual components of Fig. 4 from the main text. Fig. S8 Individual components of Fig. 5 from the main text. Fig. S9 Individual components of Fig. 6 from the main text. Fig. S10 Mutually enriched GOs from upregulated and downregulated DEGs in the same organ of winners when compared to losers of both Stenopus species. [file 12864_2026_12899_MOESM1_ESM.pdf]

Additional Information File

Transcriptomic investigation of agonistic behaviors of boxer shrimps (*Stenopus* species): Insights into the potential neural signaling roles of dopamine and acetylcholine

(Submitted to *BMC Genomics*)

Terance Ho Him Wong[a], Lai Him Chow[a], Ziwei Wu[b], Tom Kwok Lun Hui[a], Ling Ming Tsang[a]

[a] Simon F.S. Li Marine Science Laboratory, School of Life Sciences, The Chinese University of Hong Kong, Shatin, Hong Kong SAR, China

[b] Department of Microbiology and Immunology, The Peter Doherty Institute for Infection and Immunity, University of Melbourne, Victoria, Australia

Correspondence author

Email address: lmtsang@cuhk.edu.hk (L.M. Tsang)

Supplementary Information Tables

Table S1 Actual time point of cheliped loss and the final endpoint for each fighting pair of *S. hispidus* and *S. cyanoscelis*

	S. hispidus		***S. cyanoscelis***	
	Loss of cheliped (mins)	[a]Final endpoint (mins)	Loss of cheliped (mins)	[a]Final endpoint (mins)
Pair 1	32	42	15	25
Pair 2	25	35	28	38
Pair 3	19	29	24	34

[a]Final endpoint (mins) = Loss of cheliped + 10 minutes

Table S2 Full list of annotated significantly up- and downregulated differentially expressed genes (DEGs) for each pairwise comparison (i.e., winner vs control, loser vs control, and winner vs loser) of all three organs (antennae + antennules, central nervous system, and eyestalk ganglia) in *Stenopus hispidus*, *S. cyanoscelis*, and mutually in *Stenopus* https://docs.google.com/spreadsheets/d/1RiLAE4qIF-0Hf8sMuGUYdnp6csKwSSWO/edit?usp=sharing&ouid=114071563695881171971&rtpof=true&sd=true.

Table S3 Full list of significantly enriched gene ontologies (GOs) from up- and downregulated differentially expressed genes (DEGs) for each pairwise comparison (i.e., winner vs control, loser vs control, and winner vs loser) of all three organs (antennae + antennules, central nervous system, and eyestalk ganglia) in *Stenopus hispidus*, *S. cyanoscelis*, and mutually in *Stenopus* https://docs.google.com/spreadsheets/d/1PuJZzhVC73c59HHLXvEGa1SUVexh9iDL/edit?usp=sharing&ouid=114071563695881171971&rtpof=true&sd=true.

Table S4 Full list of significantly enriched Kyoto Encyclopedia of Genes and Genomes (KEGG) pathways from up- and downregulated differentially expressed genes (DEGs) for each pairwise comparison (i.e., winner vs control, loser vs control, and winner vs loser) of all three organs (antennae + antennules, central nervous system, and eyestalk ganglia) in *Stenopus hispidus*, *S. cyanoscelis*, and mutually in *Stenopus* https://docs.google.com/spreadsheets/d/1qnTVLC6LB5LVhbNoEbtu14LzqS5lM1ke/edit?usp=sharing&ouid=114071563695881171971&rtpof=true&sd=true.

Table S5 Neural signaling systems-related differentially expressed genes (DEGs) known to mediate agonistic behaviors, identified in the three organs across the three pairwise comparisons of the two studied *Stenopus* species

		S. hispidus									***S. cyanoscelis***								
		**Winner VS Control**			**Loser VS Control**			**Winner VS Loser**			**Winner VS Control**			**Loser VS Control**			**Winner VS Loser**		
Neural signaling systems		AA	CNS	EG	AA	CNS	EG	AA	CNS	EG	AA	CNS	EG	AA	CNS	EG	AA	CNS	EG
**[a]Gene set level**	Dopamine	-		+	-		+					-	+	-	-	+			
	Substance P/[c]Tachykinin	-		-			-												
	Acetylcholine	+	+	-		+			+	-		-	-	-	+	-	+	-	-
	Octopamine				+	-								+	+				
	Juvenile hormone/ [d]Methyl farnesoate				+														
	Nitric oxide		-	+						+				+					
	Serotonin							-	+	+						-			
	Glutamate		-			-			-									-	-
	GABA		+	+					-					+			-		
**[b]Gene level**	Crustacean hyperglycemic hormones		-			-													
	Crustacean hyperglycemic hormones isoform A					-													
	Crustacean hyperglycemic hormones isoform B					+								-	-		+		

[a]Gene set level refers to the neural signaling systems-related DEGs identified through GO and KEGG enrichment results, [b]Gene level refers to the DEGs that are known to mediate agonistic behaviors in pan-crustaceans but have not been assigned any (neuro)hormone-related GO or KEGG annotations, [c]Substance P homolog, [d]Juvenile hormone homolog. AA= Antennae + Antennules, CNS= Central Nervous System, EG= Eyestalk Ganglia, GABA= Gamma-aminobutyric acid. +, level elevated. -, level reduced.

Supplementary Information Figures

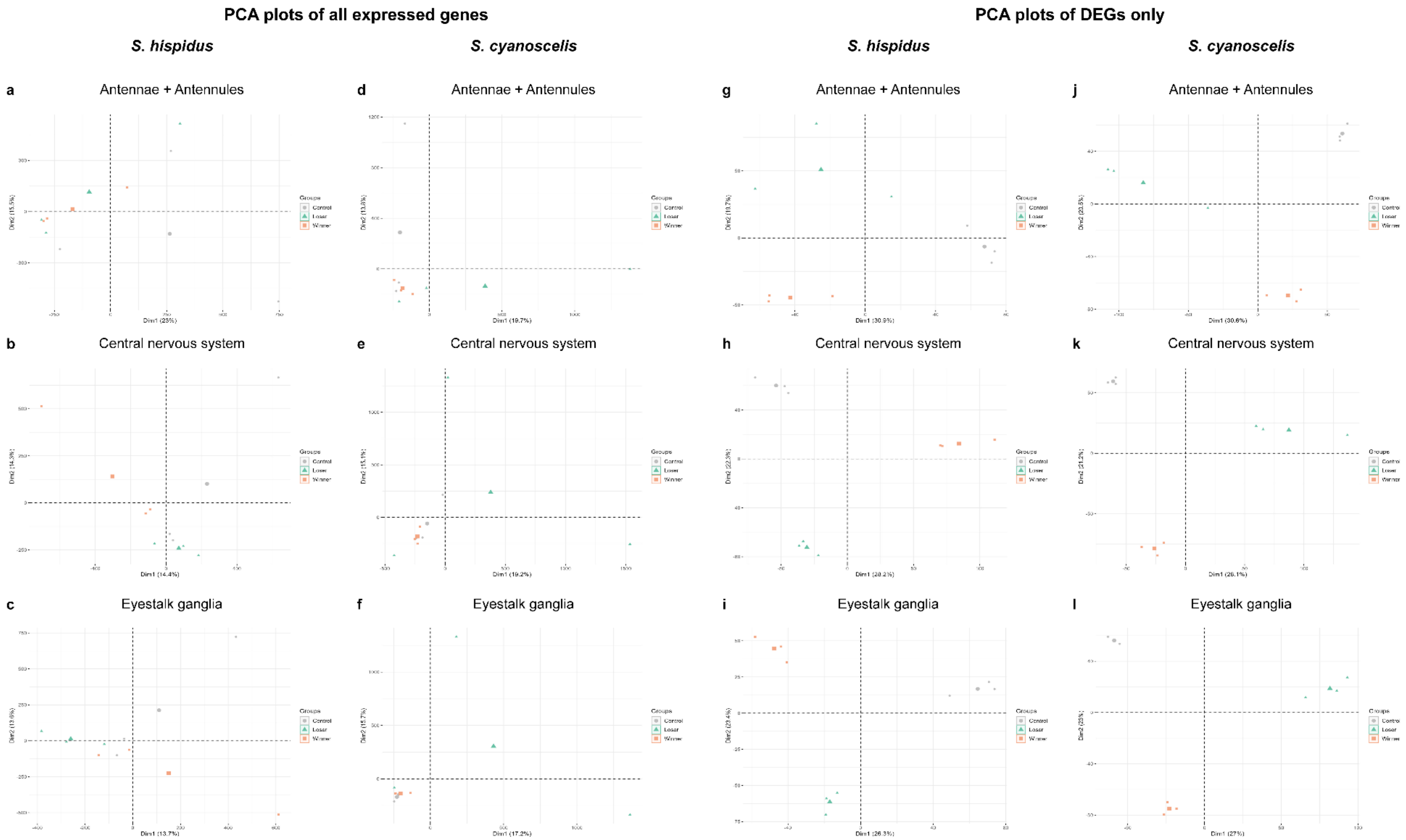

Fig. S1 Principal component analysis (PCA) based on **(a-f)** all expressed genes and **(g-l)** only the differentially expressed genes (DEGs) from the three winners, three losers, and three control individuals for the three target organs (i.e., antennae + antennules, central nervous system, and eyestalk ganglia) of *Stenopus hispidus* and *S. cyanoscelis*. Each small circle, triangle, and square in each plot represents a control, loser, and

winner individual, respectively, while the larger circle, triangle, and square represent the average expression of the three individuals for each group (i.e., control, loser, and winner). These plots suggested that fighting in *Stenopus* did not significantly alter the expression levels of most genes in the three target organs; instead, only a subset of genes showed significant differential expression (i.e., DEGs).

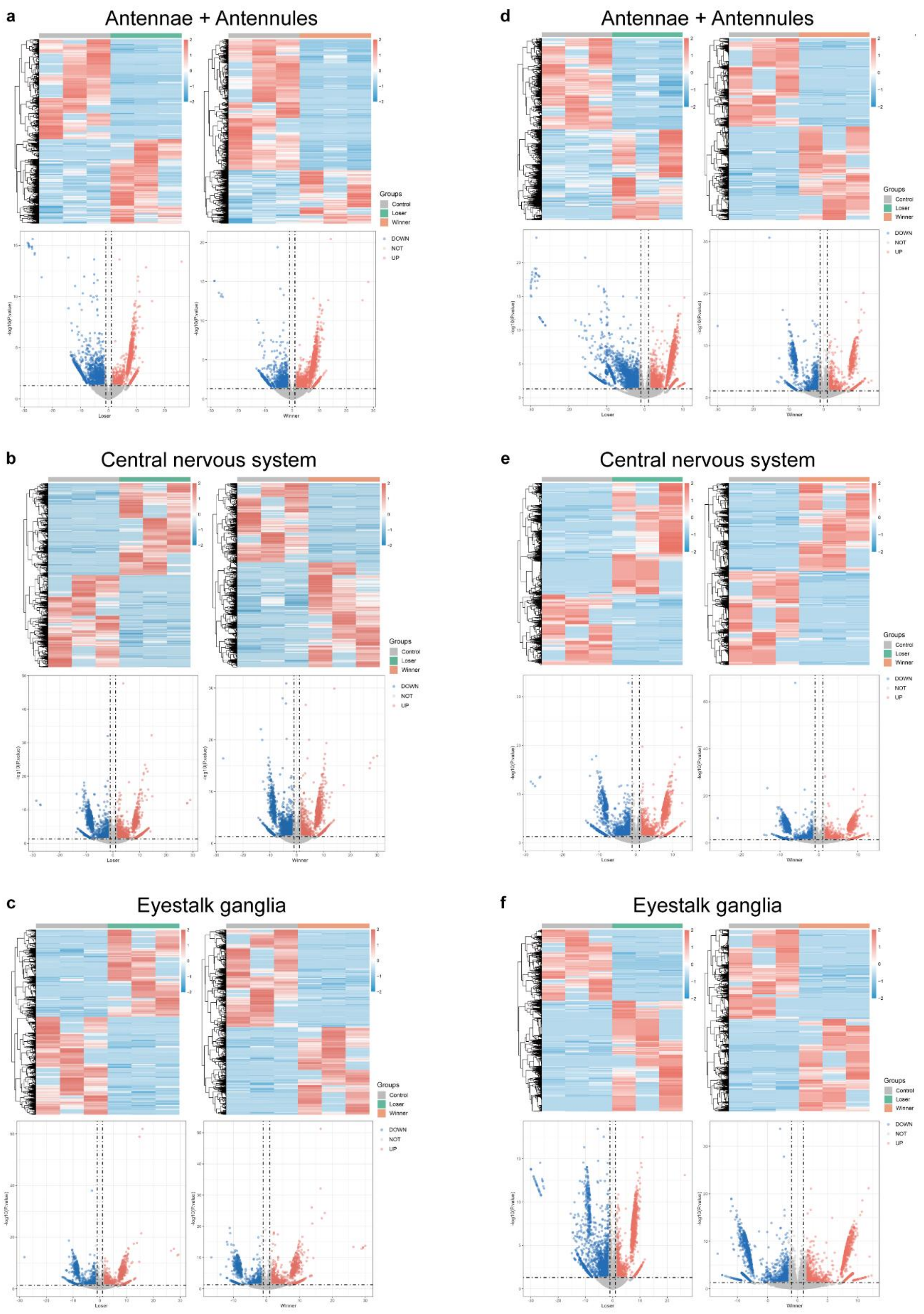

Fig. S2 Heatmap and volcano diagram showing the pattern of transcript expression and differentially expressed genes, respectively, for the three target organs (i.e., antennae + antennules, central nervous system, and eyestalk ganglia) in the winner vs control and loser vs control comparisons for **(a - c)** *S. hispidus* and **(d – f)** *S. cyanoscelis*, respectively.

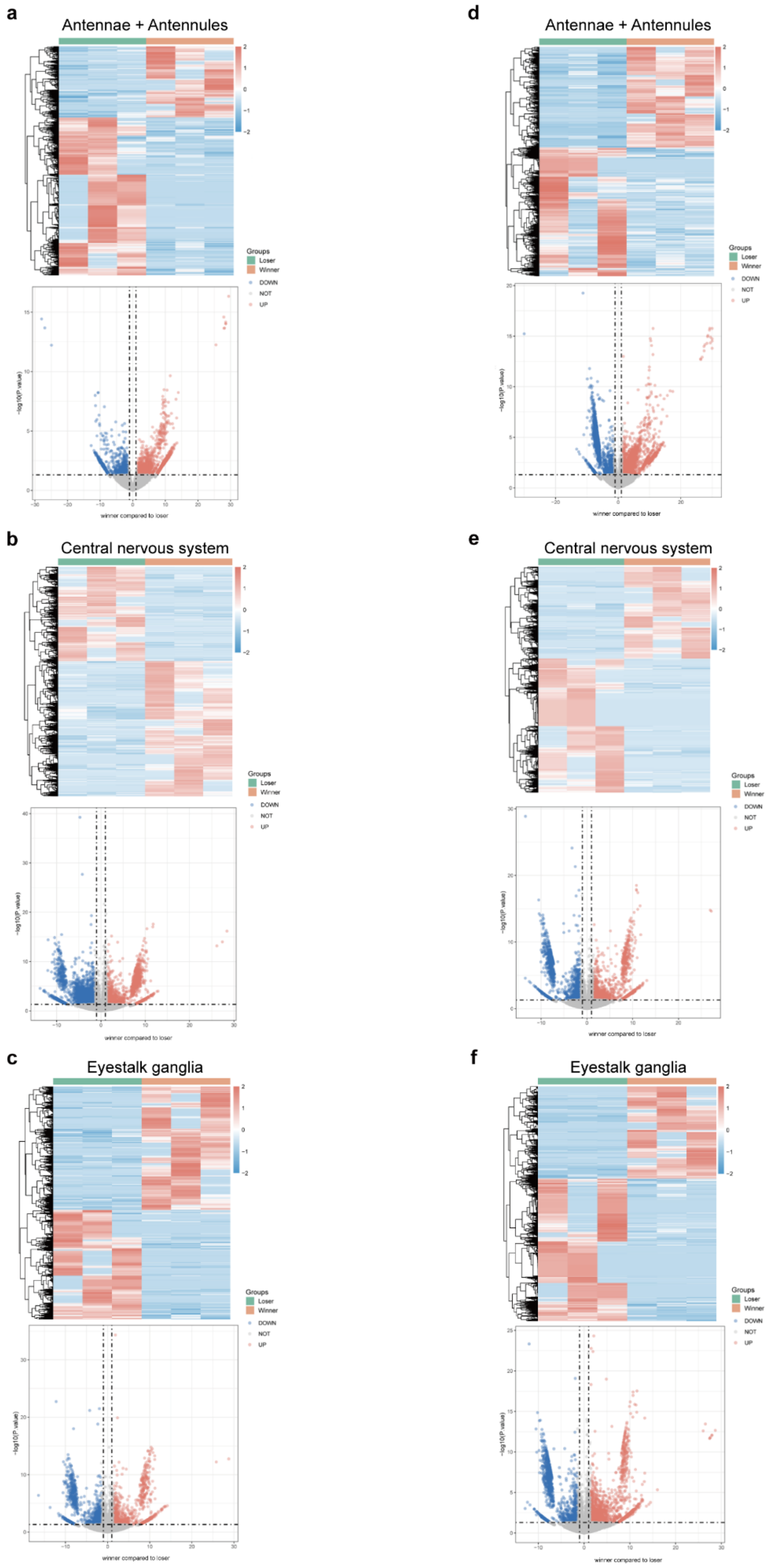

Fig. S3 Heatmap and volcano diagram showing the pattern of transcript expression and differentially expressed genes, respectively, for the three target organs (i.e., antennae + antennules, central nervous system, and eyestalk ganglia) of winners compared to losers for **(a - c)** *S. hispidus* and **(d – f)** *S. cyanoscelis*, respectively.

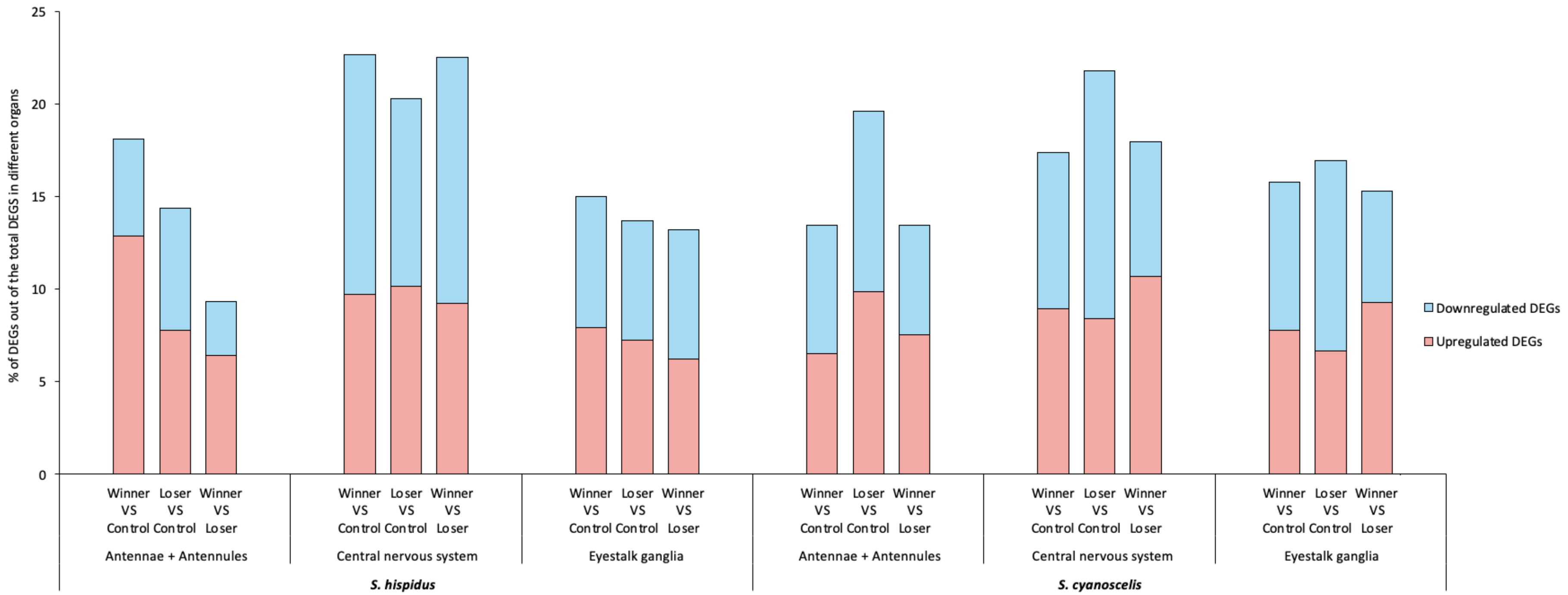

Fig. S4 Percentage of both upregulated and downregulated differentially expressed genes (DEGs) relative to the total number of DEGs in the three target organs (i.e., antennae + antennules, central nervous system, and eyestalk ganglia) for winner vs control, loser vs control, and winner vs loser comparisons in the two *Stenopus* species. Red and blue colors refer to upregulated and downregulated DEGs, respectively.

a ***S. hispidus* upregulated DEGs enriched GOs in winners and losers compared to controls**

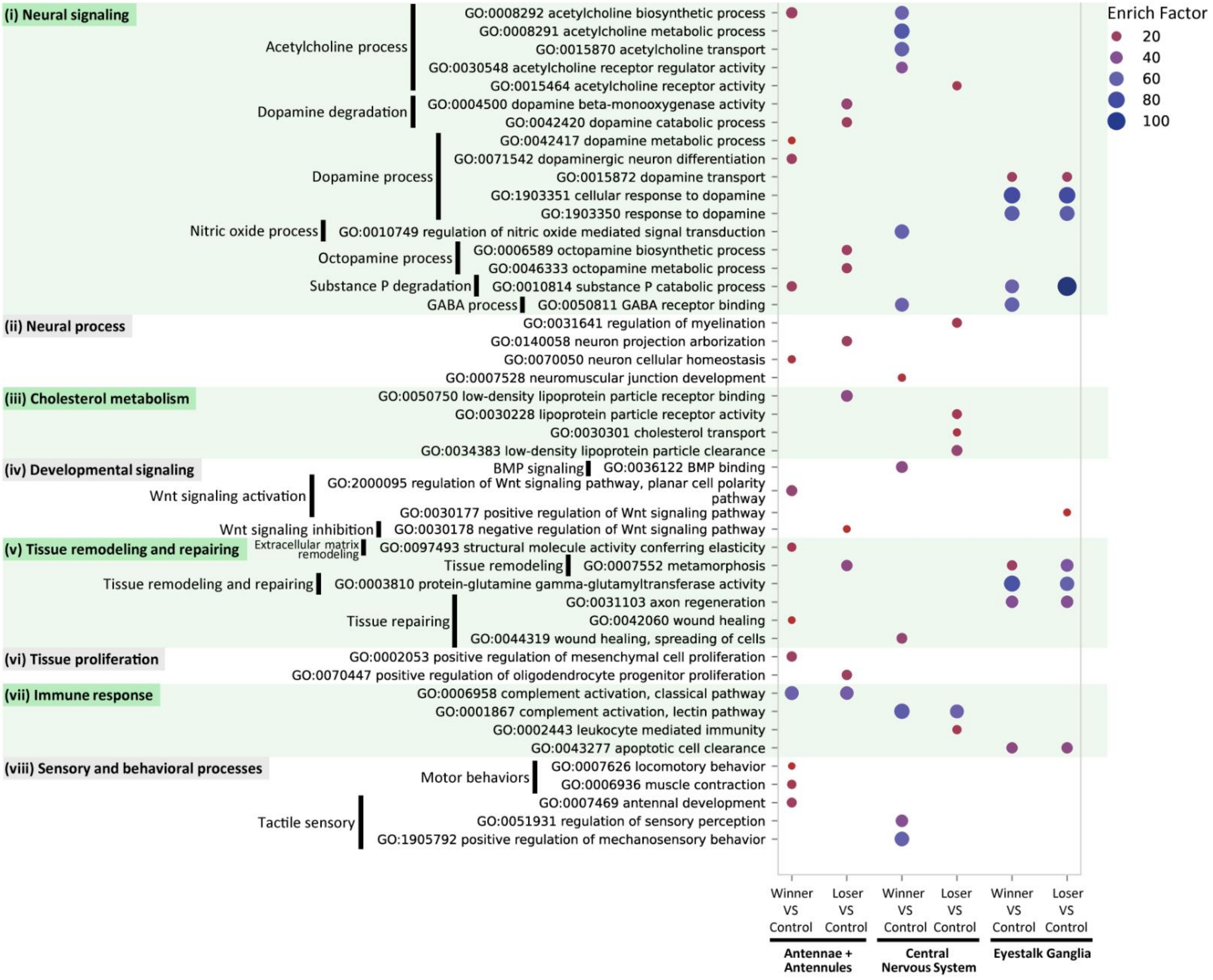

b

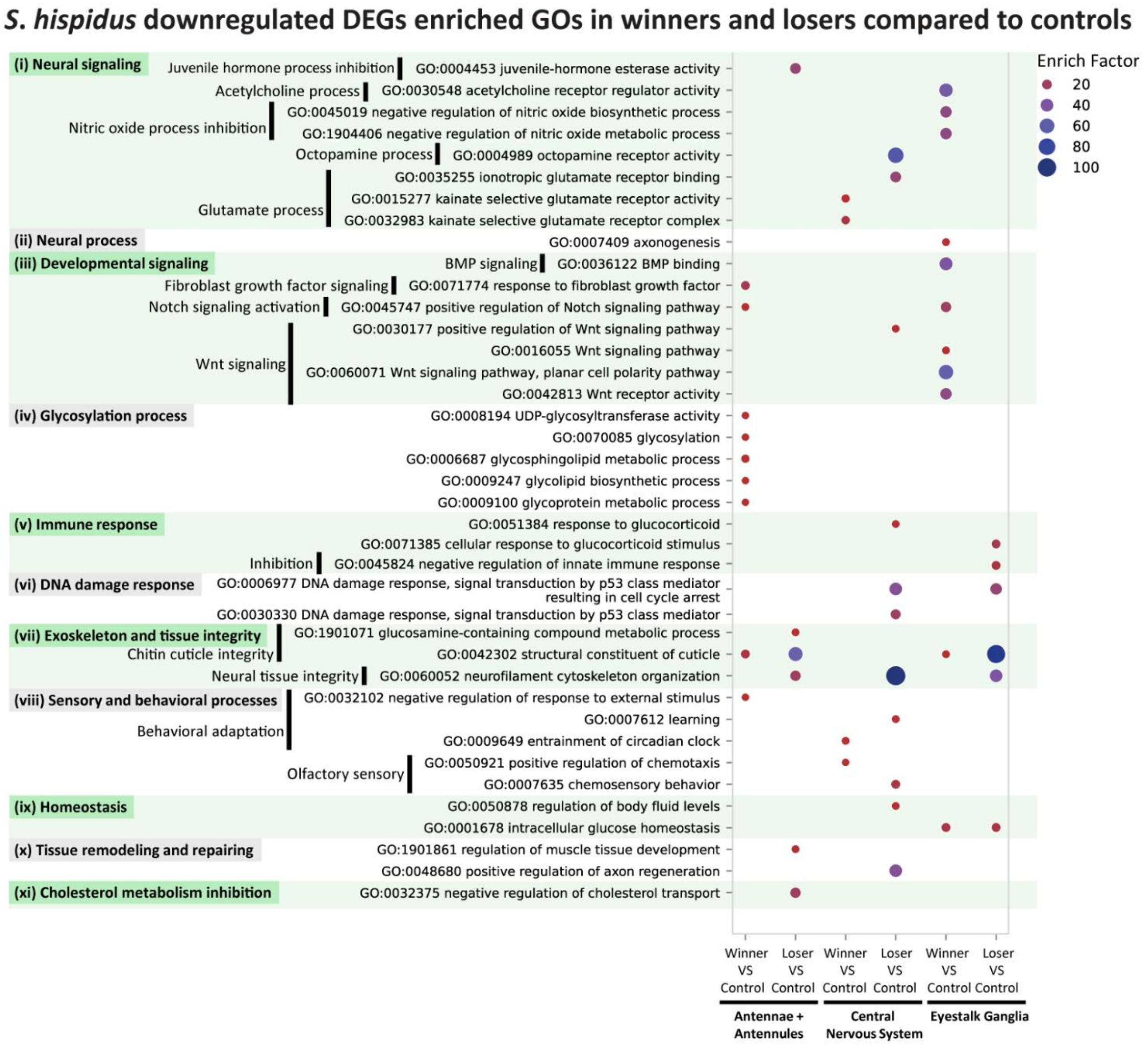

S. hispidus downregulated DEGs enriched GOs in winners and losers compared to controls
(i) Neural signaling
Juvenile hormone process inhibition
GO:0004453 juvenile-hormone esterase activity
Acetylcholine process
GO:0030548 acetylcholine receptor regulator activity
Nitric oxide process inhibition
GO:0045019 negative regulation of nitric oxide biosynthetic process
GO:1904406 negative regulation of nitric oxide metabolic process
Octopamine process
GO:0004989 octopamine receptor activity
Glutamate process
GO:0035255 ionotropic glutamate receptor binding
GO:0015277 kainate selective glutamate receptor activity
GO:0032983 kainate selective glutamate receptor complex
(ii) Neural process
GO:0007409 axonogenesis
(iii) Developmental signaling
BMP signaling
GO:0036122 BMP binding
Fibroblast growth factor signaling
GO:0071774 response to fibroblast growth factor
Notch signaling activation
GO:0045747 positive regulation of Notch signaling pathway
Wnt signaling
GO:0030177 positive regulation of Wnt signaling pathway
GO:0016055 Wnt signaling pathway
GO:0060071 Wnt signaling pathway, planar cell polarity pathway
GO:0042813 Wnt receptor activity
(iv) Glycosylation process
GO:0008194 UDP-glycosyltransferase activity
GO:0070085 glycosylation
GO:0006687 glycosphingolipid metabolic process
GO:0009247 glycolipid biosynthetic process
GO:0009100 glycoprotein metabolic process
(v) Immune response
GO:0051384 response to glucocorticoid
GO:0071385 cellular response to glucocorticoid stimulus
Inhibition
GO:0045824 negative regulation of innate immune response
(vi) DNA damage response
GO:0006977 DNA damage response, signal transduction by p53 class mediator resulting in cell cycle arrest
GO:0030330 DNA damage response, signal transduction by p53 class mediator
(vii) Exoskeleton and tissue integrity
GO:1901071 glucosamine-containing compound metabolic process
Chitin cuticle integrity
GO:0042302 structural constituent of cuticle
Neural tissue integrity
GO:0060052 neurofilament cytoskeleton organization
(viii) Sensory and behavioral processes
GO:0032102 negative regulation of response to external stimulus
Behavioral adaptation
GO:0007612 learning
GO:0009649 entrainment of circadian clock
Olfactory sensory
GO:0050921 positive regulation of chemotaxis
GO:0007635 chemosensory behavior
(ix) Homeostasis
GO:0050878 regulation of body fluid levels
GO:0001678 intracellular glucose homeostasis
(x) Tissue remodeling and repairing
GO:1901861 regulation of muscle tissue development
GO:0048680 positive regulation of axon regeneration
(xi) Cholesterol metabolism inhibition
GO:0032375 negative regulation of cholesterol transport
Enrich Factor
20
40
60
80
100
Winner VS Control
Loser VS Control
Winner VS Control
Loser VS Control
Winner VS Control
Loser VS Control
Antennae + Antennules
Central Nervous System
Eyestalk Ganglia

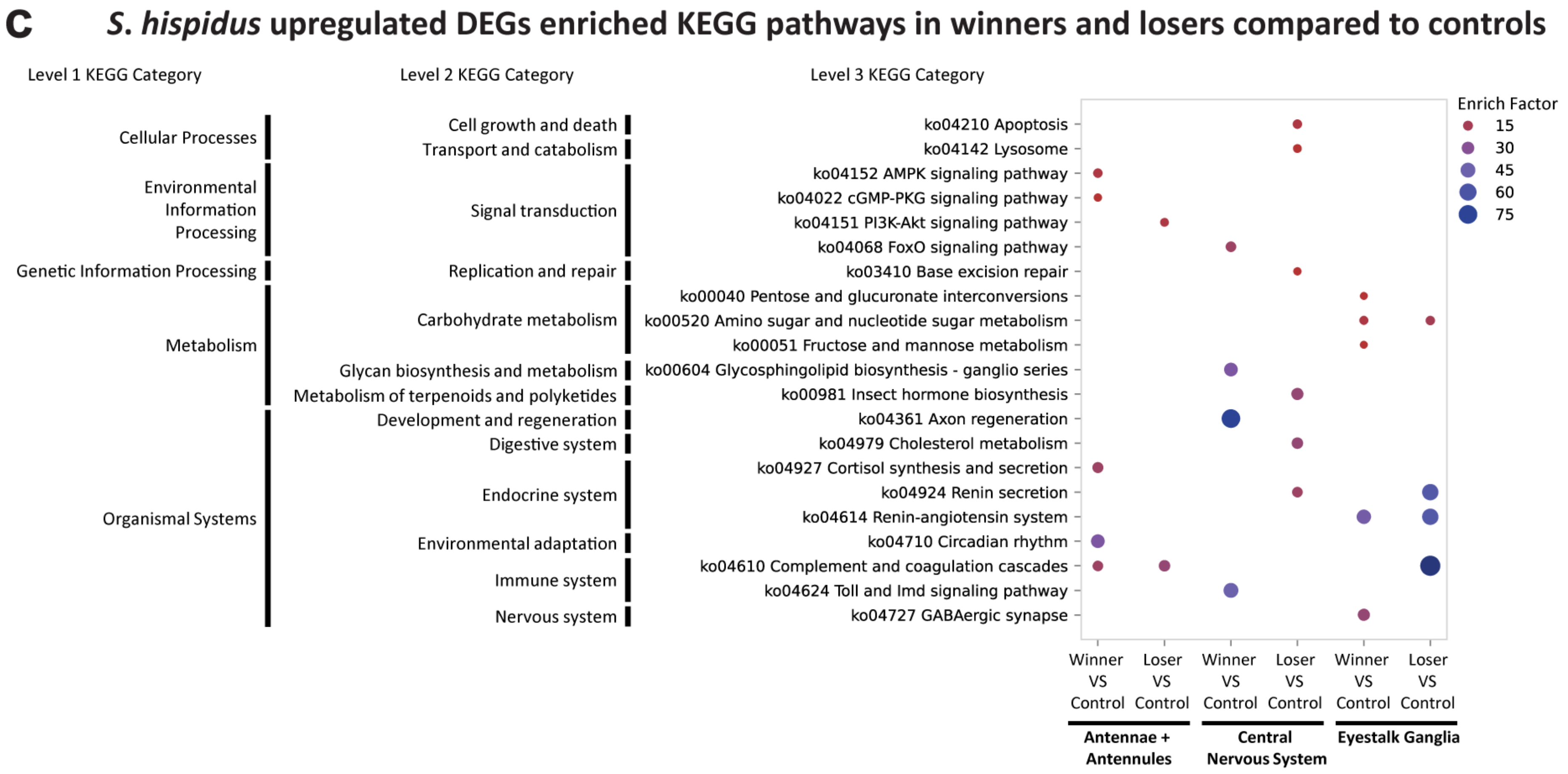
C
S. hispidus upregulated DEGs enriched KEGG pathways in winners and losers compared to controls
Level 1 KEGG Category
Level 2 KEGG Category
Level 3 KEGG Category
Cellular Processes
Environmental Information Processing
Genetic Information Processing
Metabolism
Organismal Systems
Cell growth and death
Transport and catabolism
Signal transduction
Replication and repair
Carbohydrate metabolism
Glycan biosynthesis and metabolism
Metabolism of terpenoids and polyketides
Development and regeneration
Digestive system
Endocrine system
Environmental adaptation
Immune system
Nervous system
ko04210 Apoptosis
ko04142 Lysosome
ko04152 AMPK signaling pathway
ko04022 cGMP-PKG signaling pathway
ko04151 PI3K-Akt signaling pathway
ko04068 FoxO signaling pathway
ko03410 Base excision repair
ko00040 Pentose and glucuronate interconversions
ko00520 Amino sugar and nucleotide sugar metabolism
ko00051 Fructose and mannose metabolism
ko00604 Glycosphingolipid biosynthesis - ganglio series
ko00981 Insect hormone biosynthesis
ko04361 Axon regeneration
ko04979 Cholesterol metabolism
ko04927 Cortisol synthesis and secretion
ko04924 Renin secretion
ko04614 Renin-angiotensin system
ko04710 Circadian rhythm
ko04610 Complement and coagulation cascades
ko04624 Toll and Imd signaling pathway
ko04727 GABAergic synapse
Enrich Factor
15
30
45
60
75
Winner VS Control
Loser VS Control
Winner VS Control
Loser VS Control
Winner VS Control
Loser VS Control
Antennae + Antennules
Central Nervous System
Eyestalk Ganglia

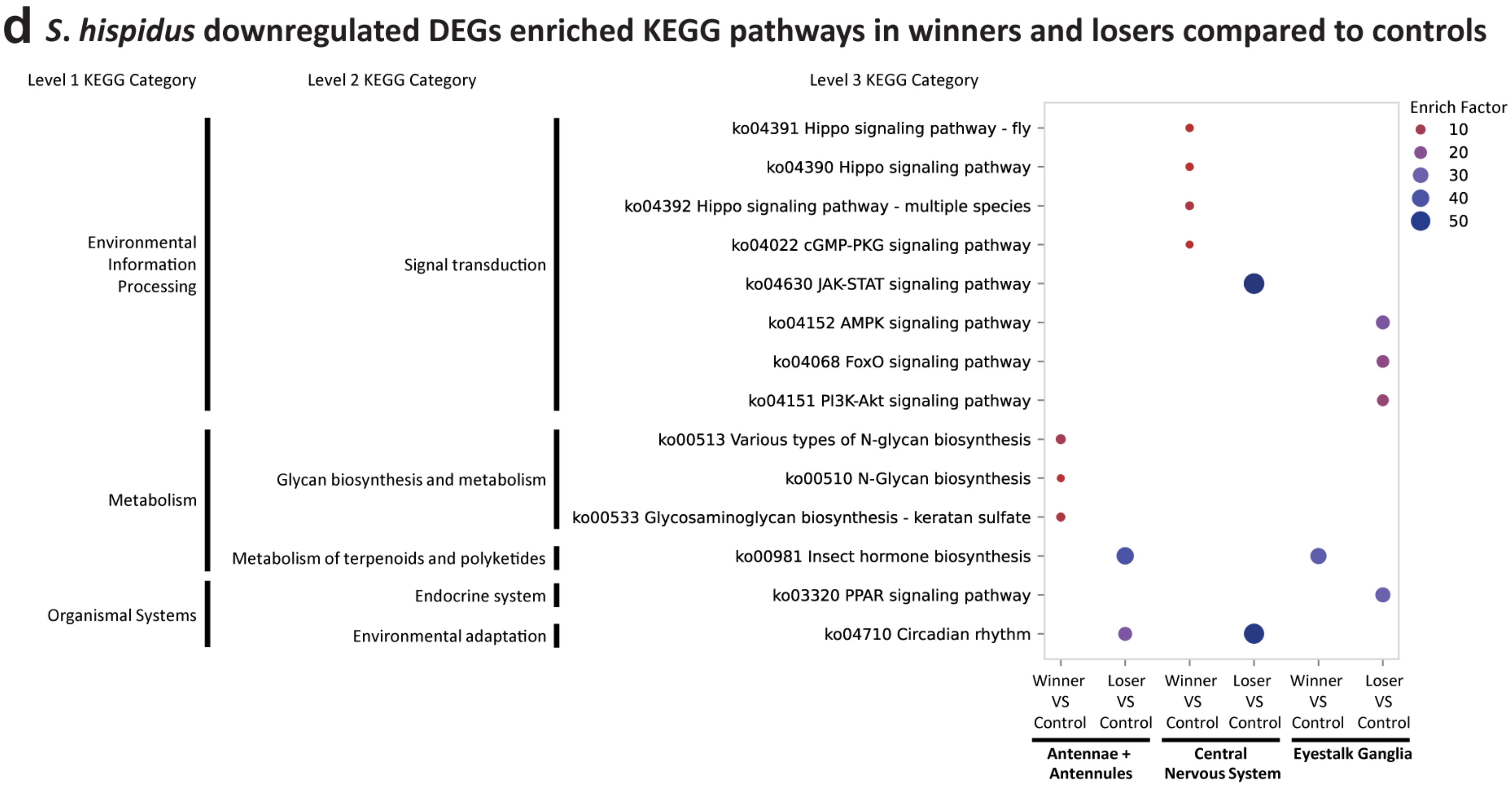

Fig. S5 Individual components of **Fig. 2** from the main text. Panels **(a)**, **(b)**, **(c)**, and **(d)** in **Fig. S5** are identical to panels **(a)**, **(b)**, **(c)**, and **(d)** in **Fig. 2.**

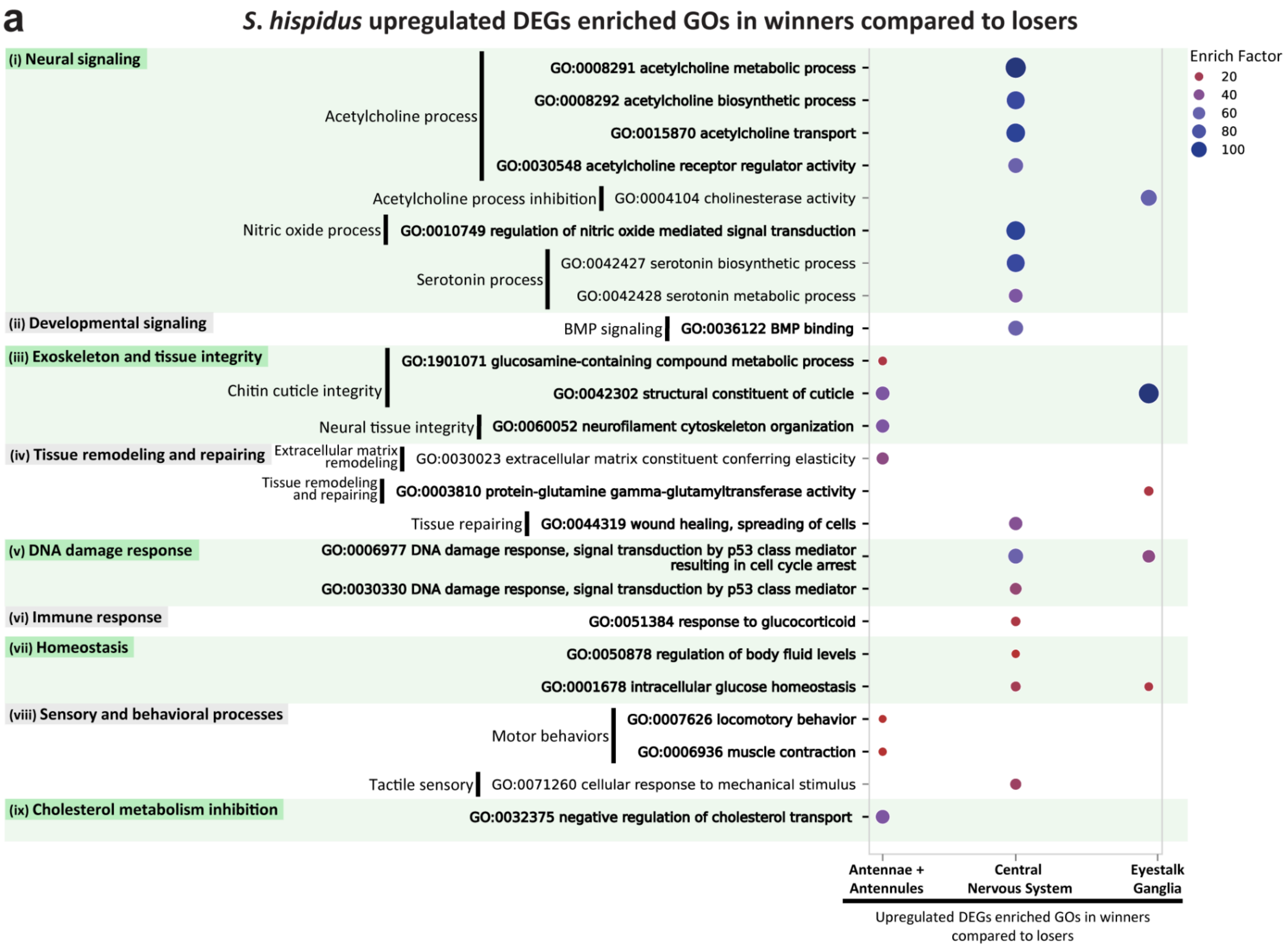
a
S. hispidus upregulated DEGs enriched GOs in winners compared to losers
Enrich Factor
20
40
60
80
100
(i) Neural signaling
Acetylcholine process
GO:0008291 acetylcholine metabolic process
GO:0008292 acetylcholine biosynthetic process
GO:0015870 acetylcholine transport
GO:0030548 acetylcholine receptor regulator activity
Acetylcholine process inhibition
GO:0004104 cholinesterase activity
Nitric oxide process
GO:0010749 regulation of nitric oxide mediated signal transduction
Serotonin process
GO:0042427 serotonin biosynthetic process
GO:0042428 serotonin metabolic process
(ii) Developmental signaling
BMP signaling
GO:0036122 BMP binding
(iii) Exoskeleton and tissue integrity
Chitin cuticle integrity
GO:1901071 glucosamine-containing compound metabolic process
GO:0042302 structural constituent of cuticle
Neural tissue integrity
GO:0060052 neurofilament cytoskeleton organization
(iv) Tissue remodeling and repairing
Extracellular matrix remodeling
GO:0030023 extracellular matrix constituent conferring elasticity
Tissue remodeling and repairing
GO:0003810 protein-glutamine gamma-glutamyltransferase activity
Tissue repairing
GO:0044319 wound healing, spreading of cells
(v) DNA damage response
GO:0006977 DNA damage response, signal transduction by p53 class mediator resulting in cell cycle arrest
GO:0030330 DNA damage response, signal transduction by p53 class mediator
(vi) Immune response
GO:0051384 response to glucocorticoid
(vii) Homeostasis
GO:0050878 regulation of body fluid levels
GO:0001678 intracellular glucose homeostasis
(viii) Sensory and behavioral processes
Motor behaviors
GO:0007626 locomotory behavior
GO:0006936 muscle contraction
Tactile sensory
GO:0071260 cellular response to mechanical stimulus
(ix) Cholesterol metabolism inhibition
GO:0032375 negative regulation of cholesterol transport
Antennae + Antennules
Central Nervous System
Eyestalk Ganglia
Upregulated DEGs enriched GOs in winners compared to losers

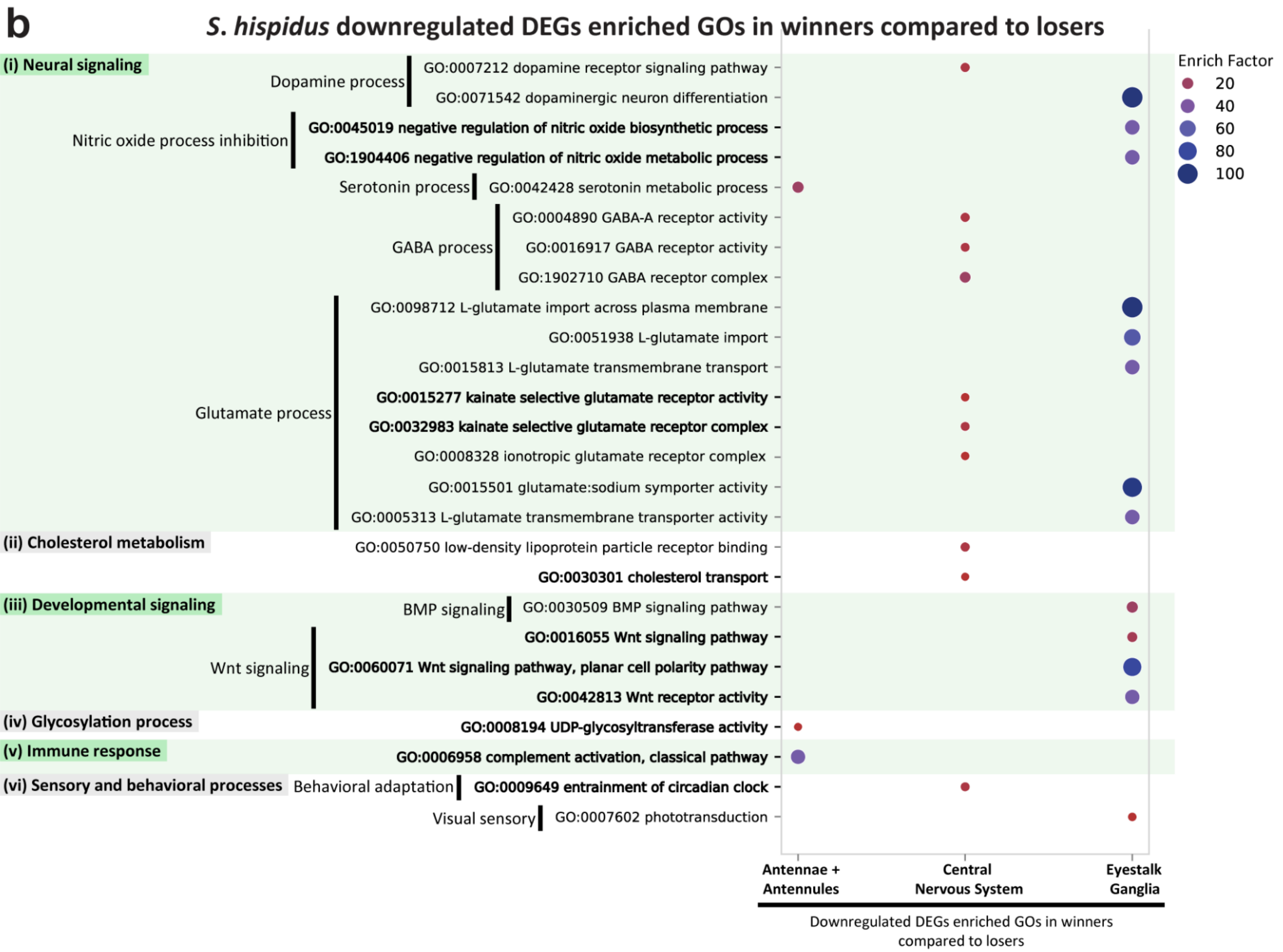

b
S. hispidus downregulated DEGs enriched GOs in winners compared to losers
(i) Neural signaling
Dopamine process
GO:0007212 dopamine receptor signaling pathway
GO:0071542 dopaminergic neuron differentiation
Nitric oxide process inhibition
GO:0045019 negative regulation of nitric oxide biosynthetic process
GO:1904406 negative regulation of nitric oxide metabolic process
Serotonin process
GO:0042428 serotonin metabolic process
GABA process
GO:0004890 GABA-A receptor activity
GO:0016917 GABA receptor activity
GO:1902710 GABA receptor complex
Glutamate process
GO:0098712 L-glutamate import across plasma membrane
GO:0051938 L-glutamate import
GO:0015813 L-glutamate transmembrane transport
GO:0015277 kainate selective glutamate receptor activity
GO:0032983 kainate selective glutamate receptor complex
GO:0008328 ionotropic glutamate receptor complex
GO:0015501 glutamate:sodium symporter activity
GO:0005313 L-glutamate transmembrane transporter activity
(ii) Cholesterol metabolism
GO:0050750 low-density lipoprotein particle receptor binding
GO:0030301 cholesterol transport
(iii) Developmental signaling
BMP signaling
GO:0030509 BMP signaling pathway
Wnt signaling
GO:0016055 Wnt signaling pathway
GO:0060071 Wnt signaling pathway, planar cell polarity pathway
GO:0042813 Wnt receptor activity
(iv) Glycosylation process
GO:0008194 UDP-glycosyltransferase activity
(v) Immune response
GO:0006958 complement activation, classical pathway
(vi) Sensory and behavioral processes
Behavioral adaptation
GO:0009649 entrainment of circadian clock
Visual sensory
GO:0007602 phototransduction
Enrich Factor
20
40
60
80
100
Antennae + Antennules
Central Nervous System
Eyestalk Ganglia
Downregulated DEGs enriched GOs in winners compared to losers

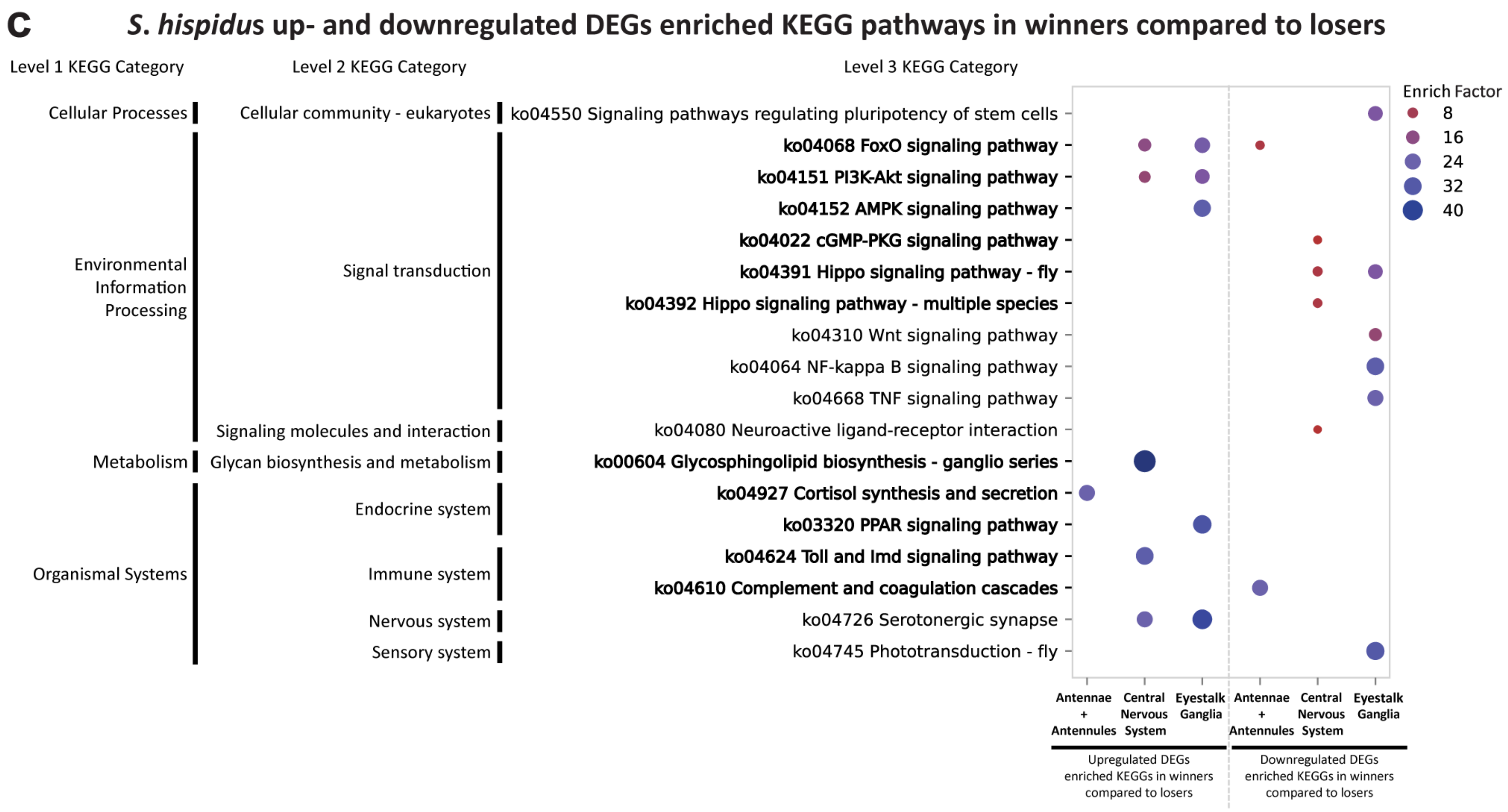

Fig. S6 Individual components of **Fig. 3** from the main text. Panels **(a)**, **(b)**, and **(c)** in **Fig. S6** are identical to panels **(a)**, **(b)**, and **(c)** in **Fig. 3.**

a

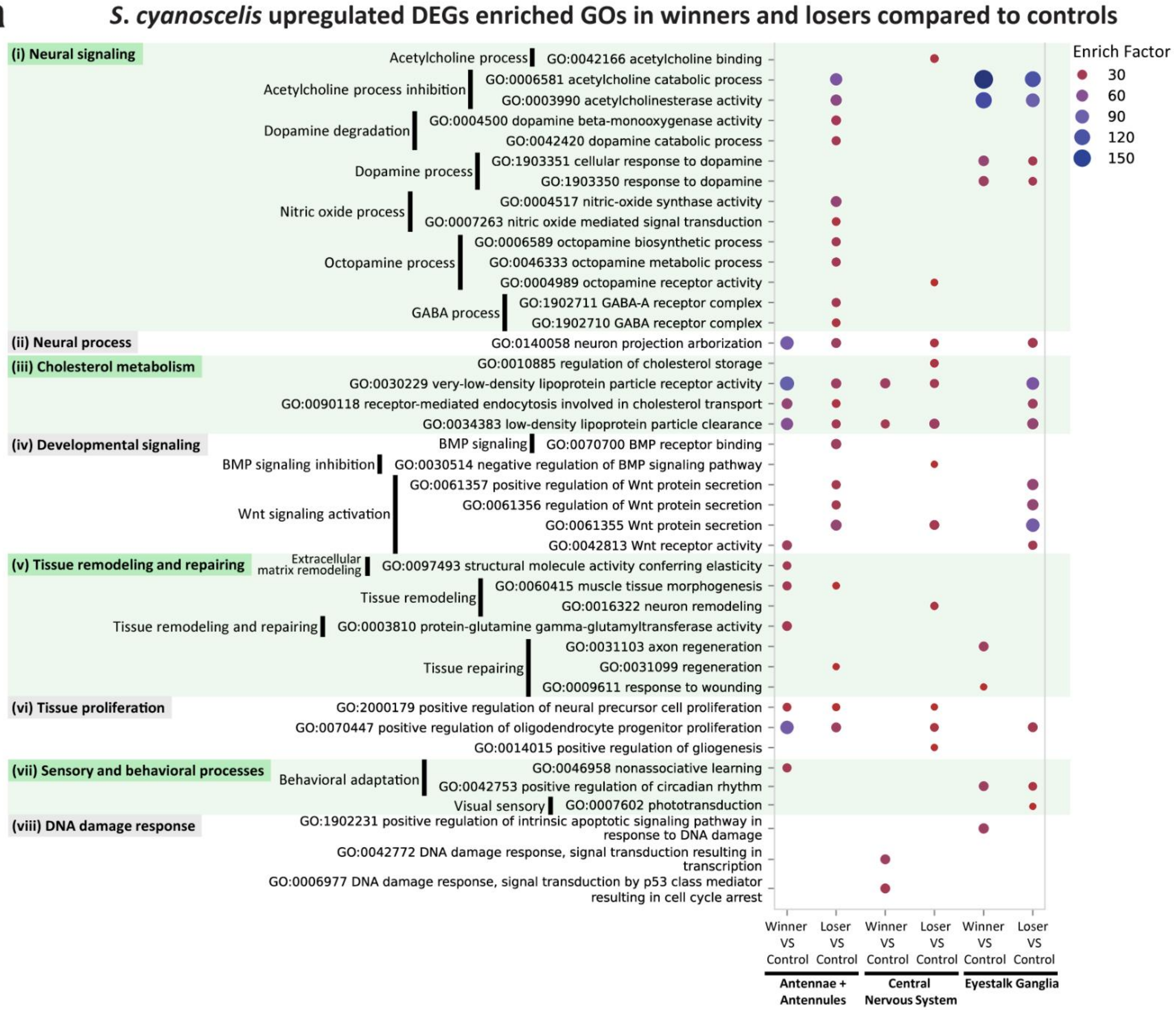

S. cyanoscelis upregulated DEGs enriched GOs in winners and losers compared to controls
Enrich Factor
30
60
90
120
150
(i) Neural signaling
Acetylcholine process
GO:0042166 acetylcholine binding
Acetylcholine process inhibition
GO:0006581 acetylcholine catabolic process
GO:0003990 acetylcholinesterase activity
Dopamine degradation
GO:0004500 dopamine beta-monooxygenase activity
GO:0042420 dopamine catabolic process
Dopamine process
GO:1903351 cellular response to dopamine
GO:1903350 response to dopamine
Nitric oxide process
GO:0004517 nitric-oxide synthase activity
GO:0007263 nitric oxide mediated signal transduction
Octopamine process
GO:0006589 octopamine biosynthetic process
GO:0046333 octopamine metabolic process
GO:0004989 octopamine receptor activity
GABA process
GO:1902711 GABA-A receptor complex
GO:1902710 GABA receptor complex
(ii) Neural process
GO:0140058 neuron projection arborization
(iii) Cholesterol metabolism
GO:0010885 regulation of cholesterol storage
GO:0030229 very-low-density lipoprotein particle receptor activity
GO:0090118 receptor-mediated endocytosis involved in cholesterol transport
GO:0034383 low-density lipoprotein particle clearance
(iv) Developmental signaling
BMP signaling
GO:0070700 BMP receptor binding
BMP signaling inhibition
GO:0030514 negative regulation of BMP signaling pathway
Wnt signaling activation
GO:0061357 positive regulation of Wnt protein secretion
GO:0061356 regulation of Wnt protein secretion
GO:0061355 Wnt protein secretion
GO:0042813 Wnt receptor activity
(v) Tissue remodeling and repairing
Extracellular matrix remodeling
GO:0097493 structural molecule activity conferring elasticity
Tissue remodeling
GO:0060415 muscle tissue morphogenesis
GO:0016322 neuron remodeling
Tissue remodeling and repairing
GO:0003810 protein-glutamine gamma-glutamyltransferase activity
Tissue repairing
GO:0031103 axon regeneration
GO:0031099 regeneration
GO:0009611 response to wounding
(vi) Tissue proliferation
GO:2000179 positive regulation of neural precursor cell proliferation
GO:0070447 positive regulation of oligodendrocyte progenitor proliferation
GO:0014015 positive regulation of gliogenesis
(vii) Sensory and behavioral processes
Behavioral adaptation
GO:0046958 nonassociative learning
GO:0042753 positive regulation of circadian rhythm
Visual sensory
GO:0007602 phototransduction
(viii) DNA damage response
GO:1902231 positive regulation of intrinsic apoptotic signaling pathway in response to DNA damage
GO:0042772 DNA damage response, signal transduction resulting in transcription
GO:0006977 DNA damage response, signal transduction by p53 class mediator resulting in cell cycle arrest
Winner VS Control
Loser VS Control
Winner VS Control
Loser VS Control
Winner VS Control
Loser VS Control
Antennae + Antennules
Central Nervous System
Eyestalk Ganglia

b ***S. cyanoscelis* downregulated DEGs enriched GOs in winners and losers compared to controls**

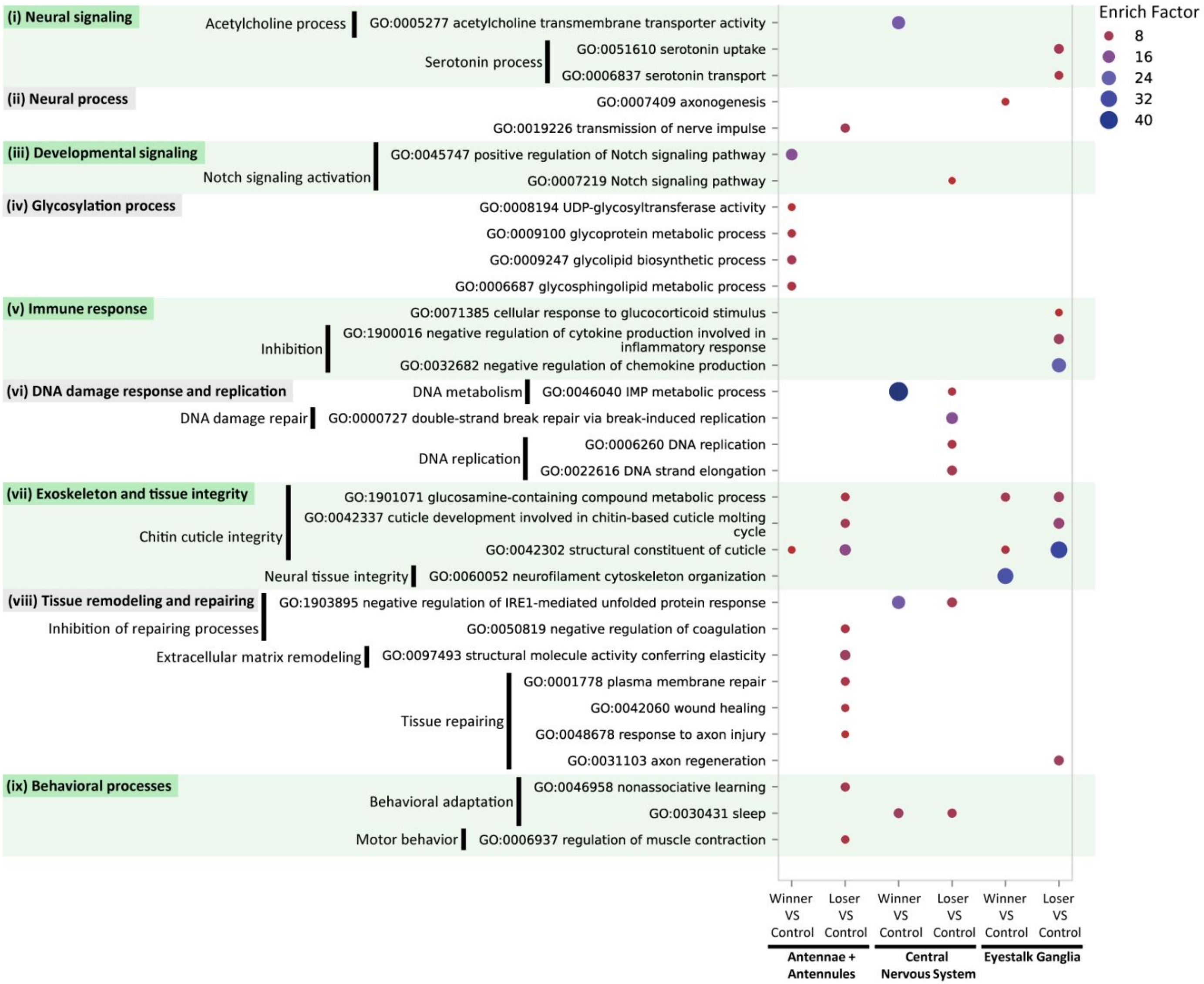

C S. cyanoscelis upregulated DEGs enriched KEGG pathways in winners and losers compared to controls

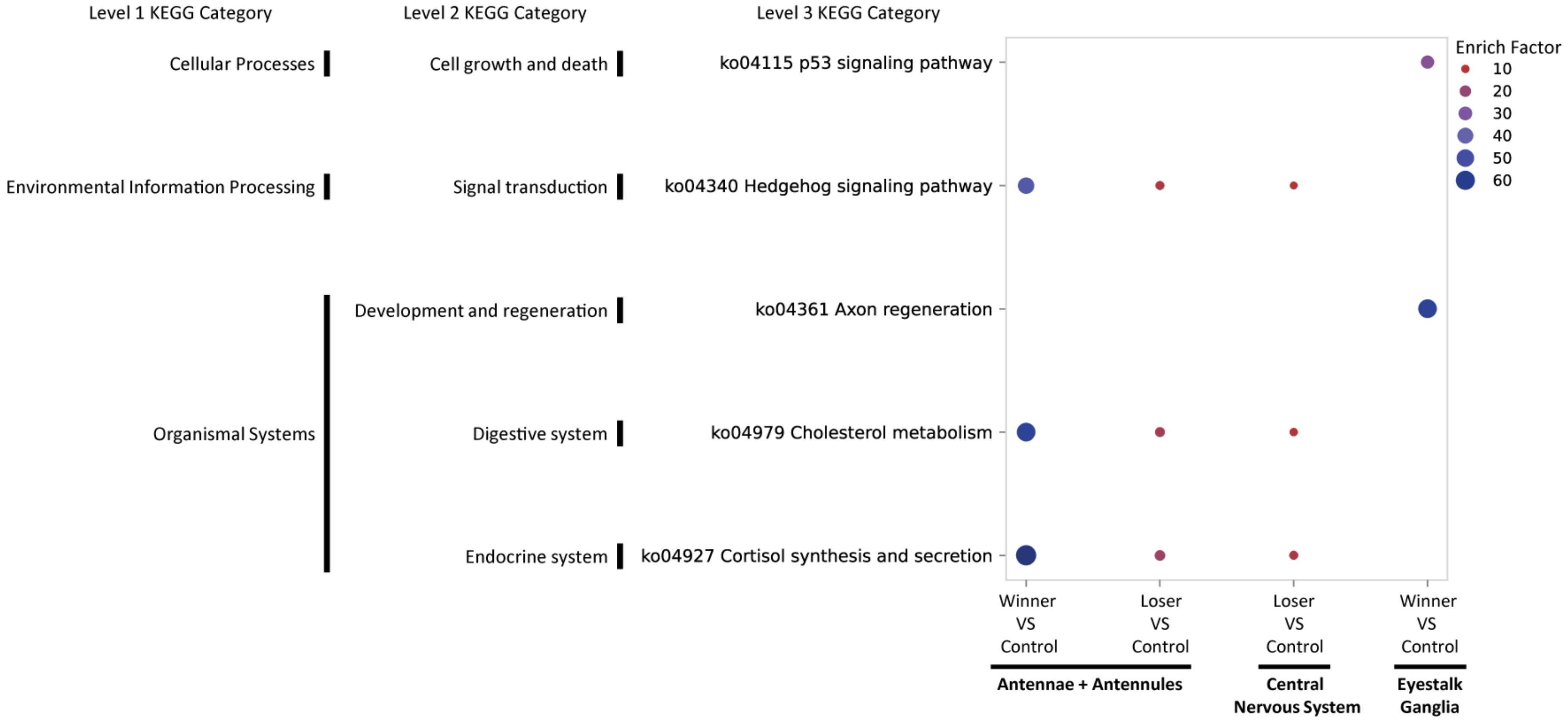

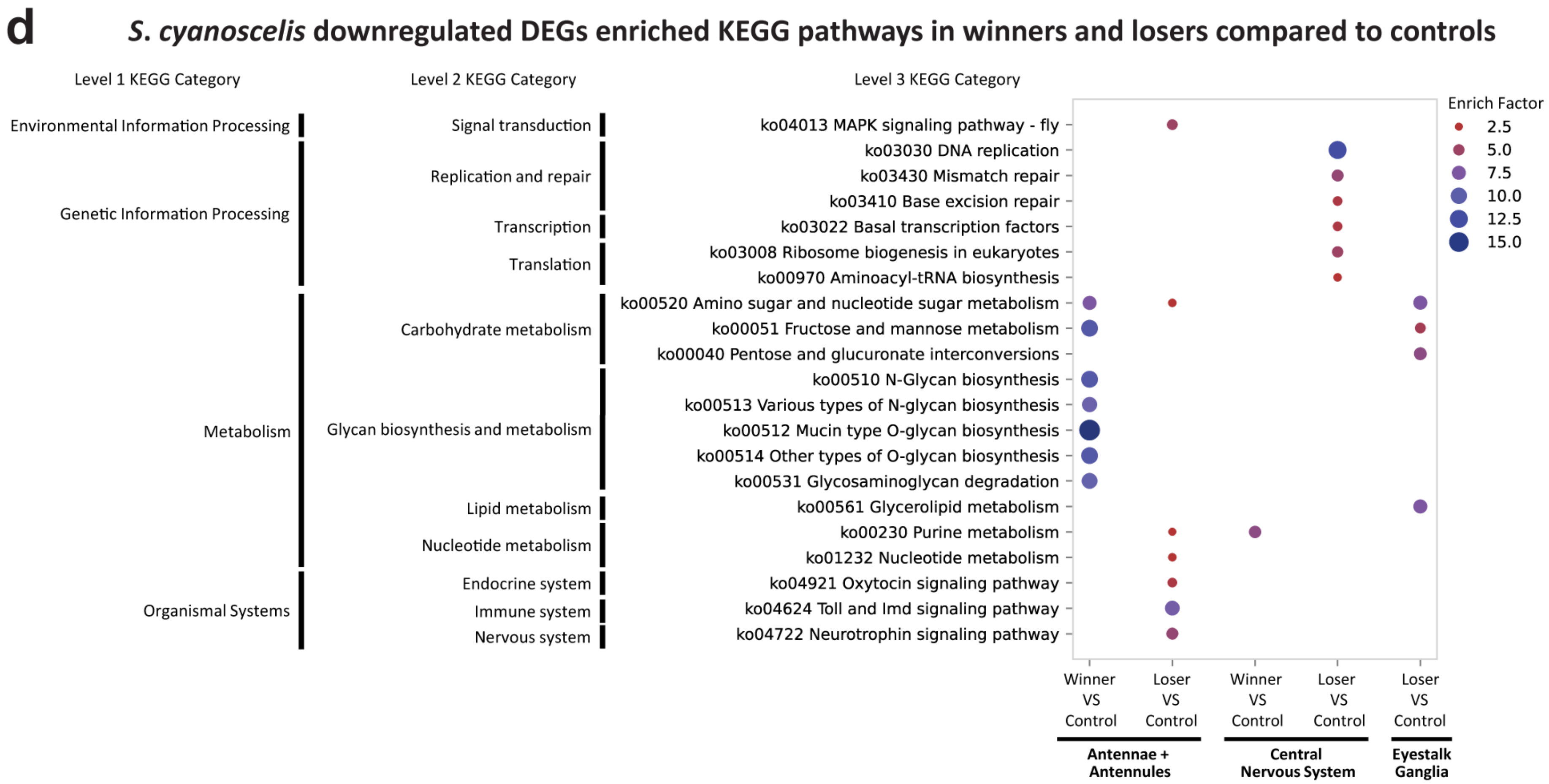

Fig. S7 Individual components of **Fig. 4** from the main text. Panels **(a)**, **(b)**, **(c)**, and **(d)** in **Fig. S7** are identical to panels **(a)**, **(b)**, **(c)**, and **(d)** in **Fig. 4.**

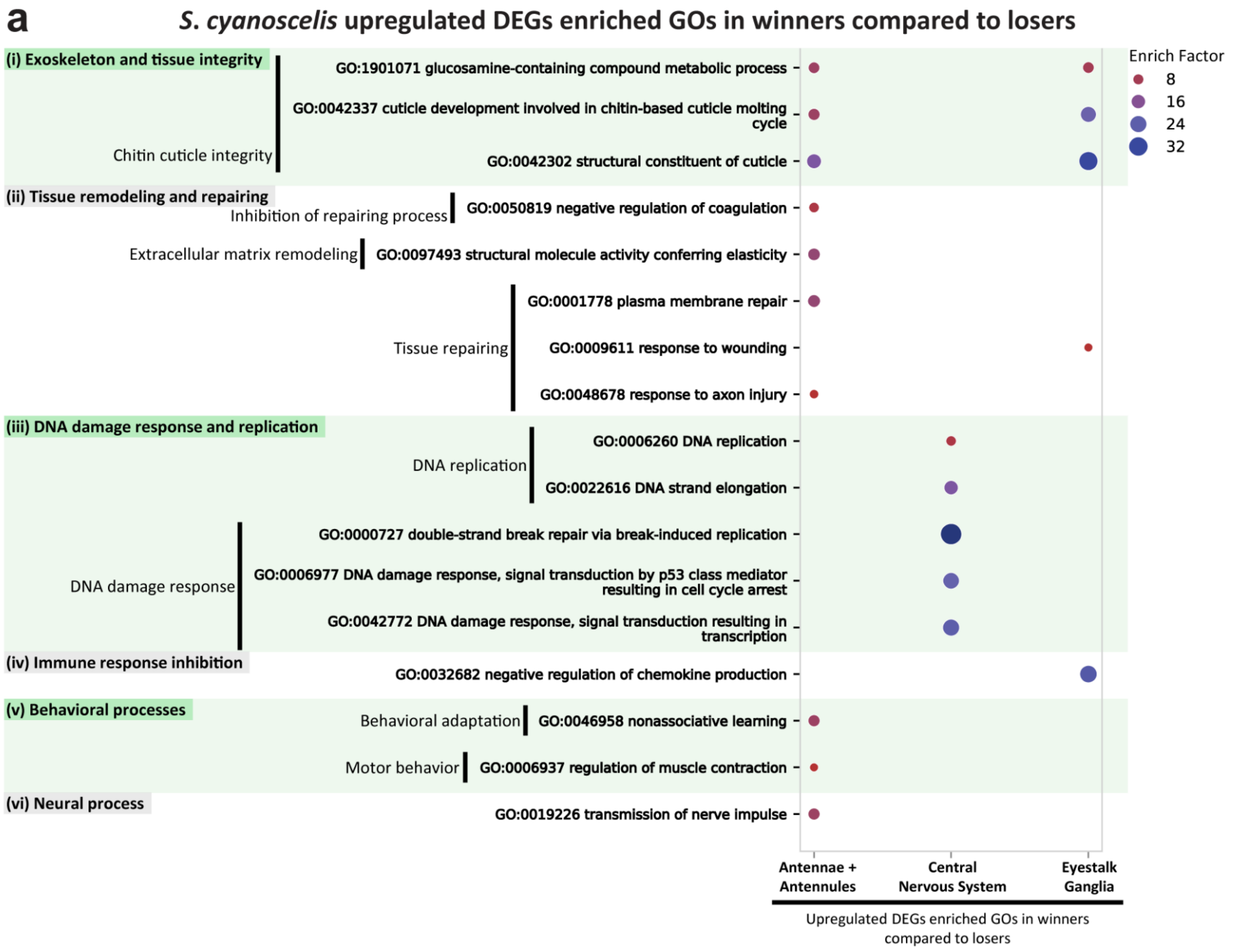

a
S. cyanoscelis upregulated DEGs enriched GOs in winners compared to losers
(i) Exoskeleton and tissue integrity
Chitin cuticle integrity
GO:1901071 glucosamine-containing compound metabolic process
GO:0042337 cuticle development involved in chitin-based cuticle molting cycle
GO:0042302 structural constituent of cuticle
(ii) Tissue remodeling and repairing
Inhibition of repairing process
GO:0050819 negative regulation of coagulation
Extracellular matrix remodeling
GO:0097493 structural molecule activity conferring elasticity
Tissue repairing
GO:0001778 plasma membrane repair
GO:0009611 response to wounding
GO:0048678 response to axon injury
(iii) DNA damage response and replication
DNA replication
GO:0006260 DNA replication
GO:0022616 DNA strand elongation
DNA damage response
GO:0000727 double-strand break repair via break-induced replication
GO:0006977 DNA damage response, signal transduction by p53 class mediator resulting in cell cycle arrest
GO:0042772 DNA damage response, signal transduction resulting in transcription
(iv) Immune response inhibition
GO:0032682 negative regulation of chemokine production
(v) Behavioral processes
Behavioral adaptation
GO:0046958 nonassociative learning
Motor behavior
GO:0006937 regulation of muscle contraction
(vi) Neural process
GO:0019226 transmission of nerve impulse
Antennae + Antennules
Central Nervous System
Eyestalk Ganglia
Upregulated DEGs enriched GOs in winners compared to losers
Enrich Factor
8
16
24
32

b

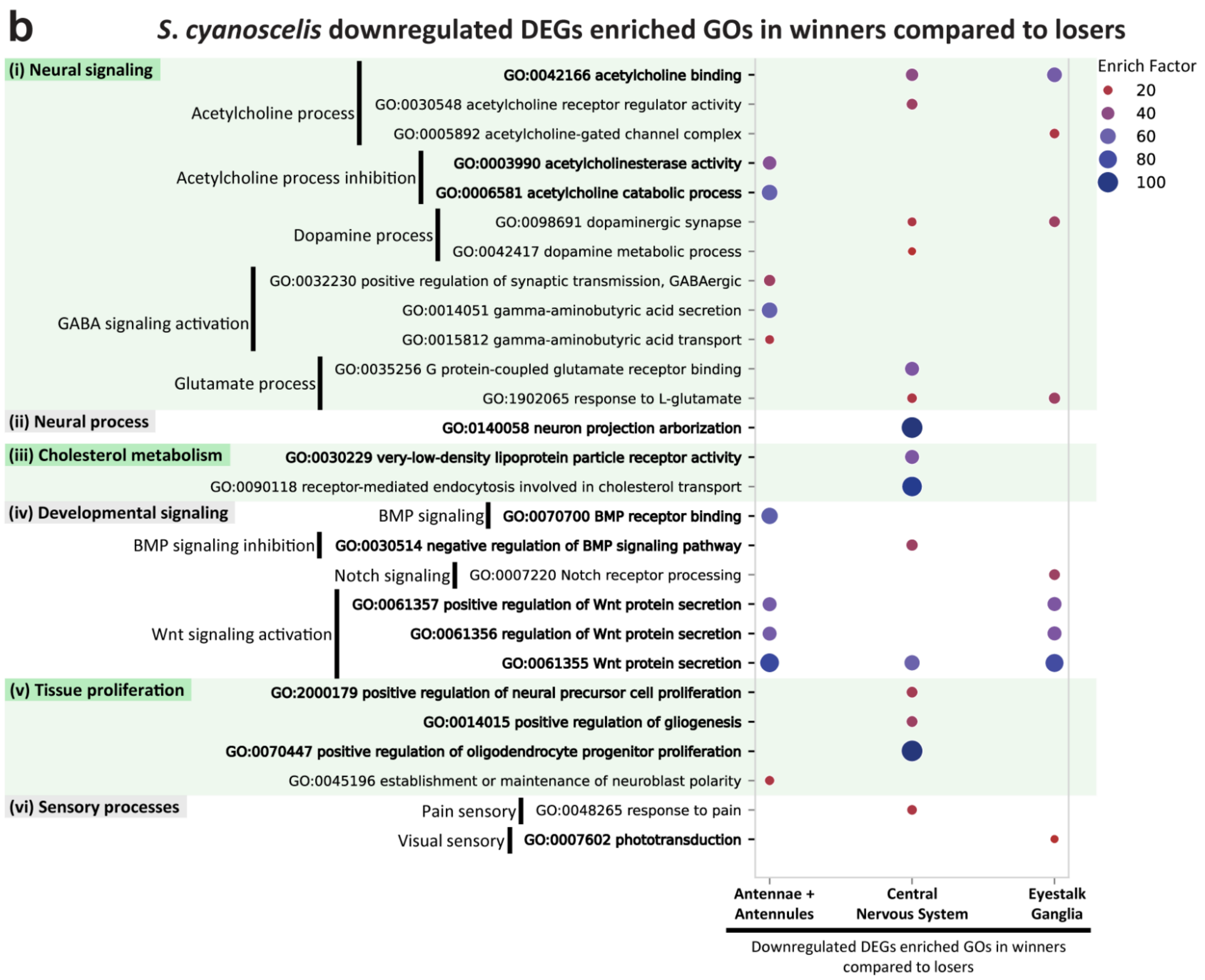
S. cyanoscelis downregulated DEGs enriched GOs in winners compared to losers
(i) Neural signaling
Acetylcholine process
GO:0042166 acetylcholine binding
GO:0030548 acetylcholine receptor regulator activity
GO:0005892 acetylcholine-gated channel complex
Acetylcholine process inhibition
GO:0003990 acetylcholinesterase activity
GO:0006581 acetylcholine catabolic process
Dopamine process
GO:0098691 dopaminergic synapse
GO:0042417 dopamine metabolic process
GABA signaling activation
GO:0032230 positive regulation of synaptic transmission, GABAergic
GO:0014051 gamma-aminobutyric acid secretion
GO:0015812 gamma-aminobutyric acid transport
Glutamate process
GO:0035256 G protein-coupled glutamate receptor binding
GO:1902065 response to L-glutamate
(ii) Neural process
GO:0140058 neuron projection arborization
(iii) Cholesterol metabolism
GO:0030229 very-low-density lipoprotein particle receptor activity
GO:0090118 receptor-mediated endocytosis involved in cholesterol transport
(iv) Developmental signaling
BMP signaling
GO:0070700 BMP receptor binding
BMP signaling inhibition
GO:0030514 negative regulation of BMP signaling pathway
Notch signaling
GO:0007220 Notch receptor processing
Wnt signaling activation
GO:0061357 positive regulation of Wnt protein secretion
GO:0061356 regulation of Wnt protein secretion
GO:0061355 Wnt protein secretion
(v) Tissue proliferation
GO:2000179 positive regulation of neural precursor cell proliferation
GO:0014015 positive regulation of gliogenesis
GO:0070447 positive regulation of oligodendrocyte progenitor proliferation
GO:0045196 establishment or maintenance of neuroblast polarity
(vi) Sensory processes
Pain sensory
GO:0048265 response to pain
Visual sensory
GO:0007602 phototransduction
Enrich Factor
20
40
60
80
100
Antennae + Antennules
Central Nervous System
Eyestalk Ganglia
Downregulated DEGs enriched GOs in winners compared to losers

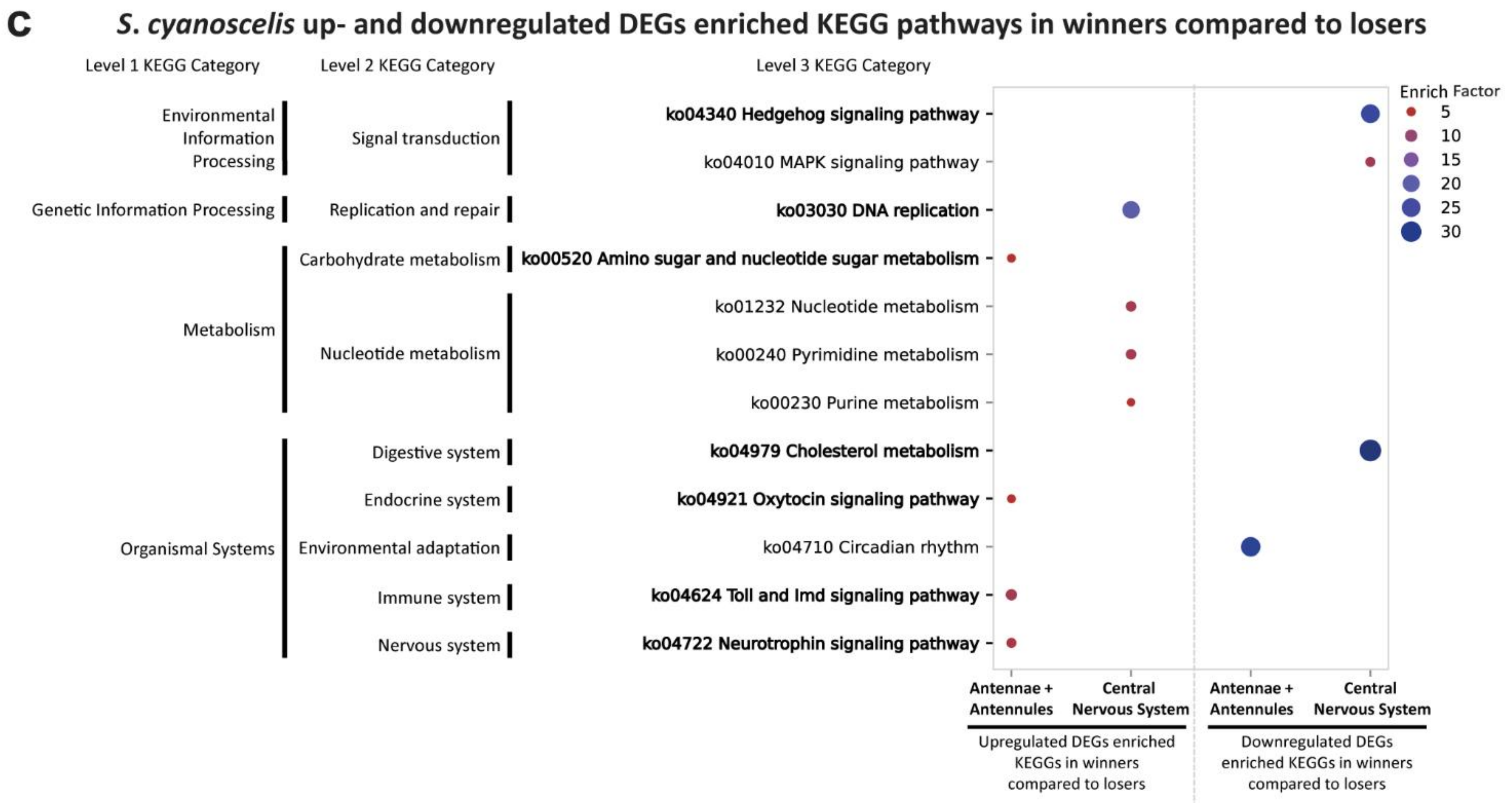

Fig. S8 Individual components of **Fig. 5** from the main text. Panels **(a)**, **(b)**, and **(c)** in **Fig. S8** are identical to panels **(a)**, **(b)**, and **(c)** in **Fig. 5.**

a

Mutually enriched GOs from up- and downregulated DEGs of winners compared to controls and losers compared to controls for the two *Stenopus* species

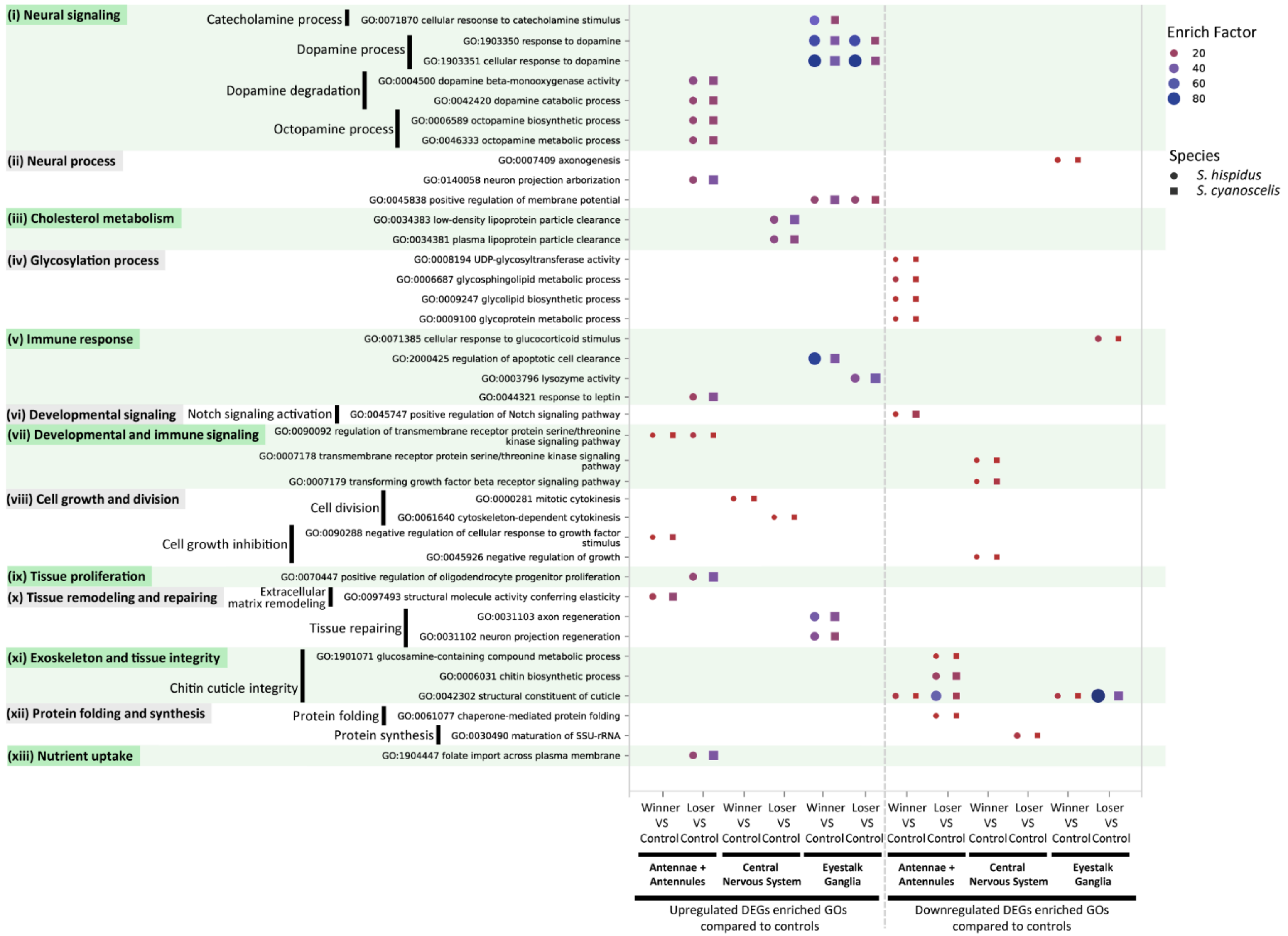

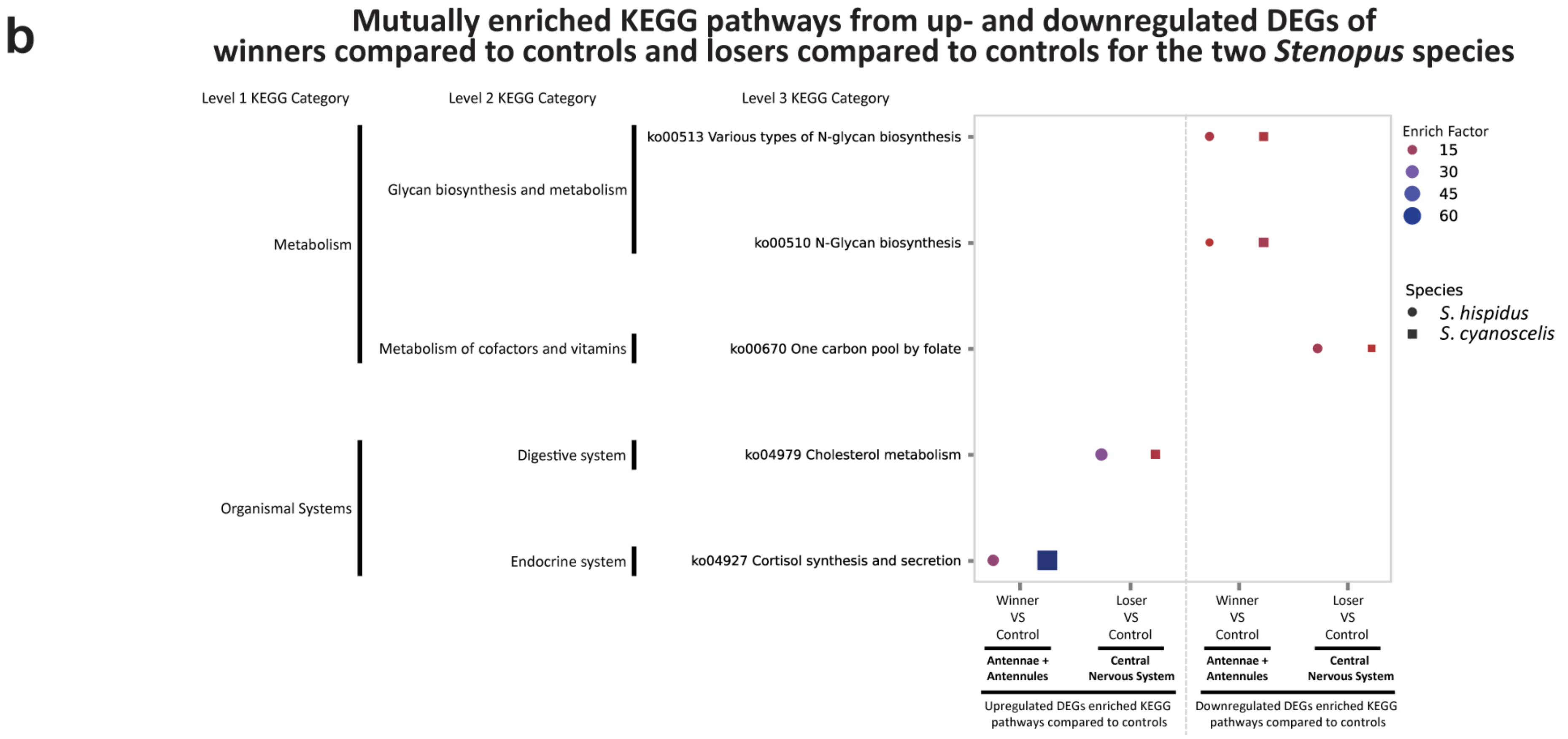

Fig. S9 Individual components of **Fig. 6** from the main text. Panels **(a)** and **(b)** in **Fig. S9** are identical to panels **(a)** and **(b)** in **Fig. 6.**

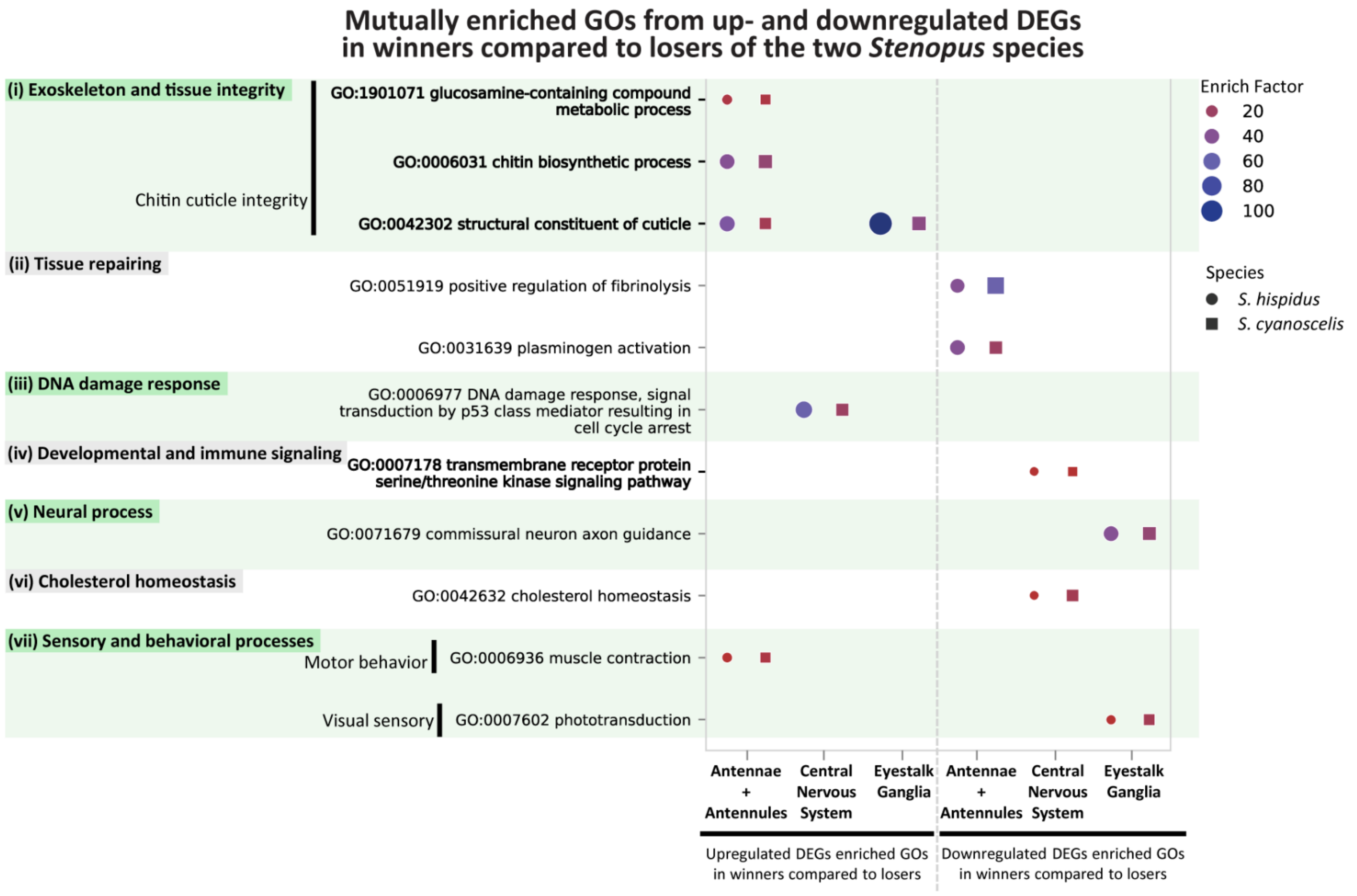

Fig. S10 Mutually enriched GOs from upregulated and downregulated DEGs in the same organ (i.e., antennae + antennules, central nervous system, and eyestalk ganglia) of winners when compared to losers of both *Stenopus* species. All enriched GO terms are statistically significant at $p < 0.05$ and were grouped according to their typical functions. Individually bolded GOs indicate that their DEGs were statistically significant in at least one treatment vs control comparison.
